# Sliding over the Blocks in Enzyme-Free RNA Copying – One-Pot Primer Extension in Ice

**DOI:** 10.1371/journal.pone.0075617

**Published:** 2013-09-18

**Authors:** Philipp M. G. Löffler, Joost Groen, Mark Dörr, Pierre-Alain Monnard

**Affiliations:** 1 Department of Physics, Chemistry and Pharmaceutical Sciences, University of Southern Denmark, Odense, Denmark; 2 Institute for Molecules and Materials, Radboud University Nijmegen, Nijmegen, Netherlands; 3 Department of Biotechnology & Enzyme Catalysis, Ernst-Moritz-Arndt University of Greifswald, Greifswald, Germany; Ben-Gurion University, Israel

## Abstract

Template-directed polymerization of RNA in the absence of enzymes is the basis for an information transfer in the ‘RNA-world’ hypothesis and in novel nucleic acid based technology. Previous investigations established that only cytidine rich strands are efficient templates in bulk aqueous solutions while a few specific sequences completely block the extension of hybridized primers. We show that a eutectic water/ice system can support Pb^2+^/Mg^2+^-ion catalyzed extension of a primer across such sequences, i.e. AA, AU and AG, in a one-pot synthesis. Using mixtures of imidazole activated nucleotide 5′-monophosphates, the two first “blocking” residues could be passed during template-directed polymerization, i.e., formation of triply extended products containing a high fraction of faithful copies was demonstrated. Across the AG sequence, a mismatch sequence was formed in similar amounts to the correct product due to U·G wobble pairing. Thus, the template-directed extension occurs both across pyrimidine and purine rich sequences and insertions of pyrimidines did not inhibit the subsequent insertions. Products were mainly formed with 2′-5′-phosphodiester linkages, however, the abundance of 3′–5′-linkages was higher than previously reported for pyrimidine insertions. When enzyme-free, template-directed RNA polymerization is performed in a eutectic water ice environment, various intrinsic reaction limitations observed in bulk solution can then be overcome.

## Introduction

The understanding of RNA polymerization chemistry in aqueous environments, in particular the template-directed copying process, is central to the investigation of the spontaneous emergence of self-replicating nucleic acids as proposed in the ‘RNA world’ hypothesis [1,2]. Successful model systems for enzyme-free nucleic acid information transfer will also have applications in novel approaches to biosensing, genotyping and sequencing [3]. As one model system, template-directed primer extension reactions performed in aqueous solution have been studied thoroughly.

Many different approaches have been reported, such as self-templating, self-priming RNA hairpins [4-6] or single stranded RNA or DNA templates and short primer sequences carrying a reporter dye [7]. Extensive reviews of this body of work can be found in the literature [8-10]. In this framework, the presence of C- and G-rich templates allows effective primer elongations with high fidelity [4,5,11]. Various DNA (RNA) template sequence motifs, however, were reported to completely block spontaneous primer elongation: AA, AT (AU), AG, TA (UA), and TT (UU). Only single A- or T (U) -residues followed by C-residues could be copied in these experiments [6]. Most notably, activated uridine monomers do not oligomerize efficiently bulk aqueous solution in the presence nor absence of a polyadenylic template [12].

The work reported herein builds upon previous observations that U monomers self-condensate efficiently when reaction mixtures are incubated below their freezing temperature, but above their eutectic point, in the presence of low concentrations of lead and magnesium cations [13]. This environment, called eutectic water-ice phase, hosts the reaction in a semi-compartmentalizing µm-scale network of up-concentrated solution channels within a solid ice matrix. In contrast to bulk solution reactions, where the condensation of purine is favored over that of pyrimidine monomers, all canonical ribonucleotides polymerize equally effectively in the eutectic phase [14] and furthermore polymerization exclusively occurs if the eutectic water-ice phase is formed, i.e., the majority of the water is frozen [13]. Finally, the extension of a RNA hairpin could be achieved for every ribonucleotide individually, and the highest monomer elongation rates were observed when the monomer was inserted opposite to its cognate template residue [15].

In recent publications, very efficient aqueous phase template-directed primer elongations were reported, using downstream binding strands [16,17], or an alternative semi-reversible amide-backbone chemistry [18], but these approaches either require replenishing the substrate pool and exchanging downstream binding strands or a different backbone-chemistry – i.e., reductive amination, respectively. The present work aims at investigating “one-pot” conditions with RNA monomers, i.e. using mixtures of activated monomers supplied at once.

In the herein reported primer extension assays, nucleoside 5′-phosphoroimidazolides (ImpN) or nucleoside 5′-phosphoro(2-methyl) imidazolides (2-MeImpN) have been employed as activated monomers species. The 2′- or 3′-OH of a primer reacts with these monomers by a nucleophilic attack at the phosphorous atom, catalyzed by a mixture of Mg^2+^ and Pb^2+^ ions [13]. The subsequent elimination of the activating group then forms either a 2′-5′- or 3′–5′-phosphodiester bond [19,20]. RNA polymerization is catalyzed by various divalent metal ions [21], but particularly well by lead(II) [22].

**Figure 1:**
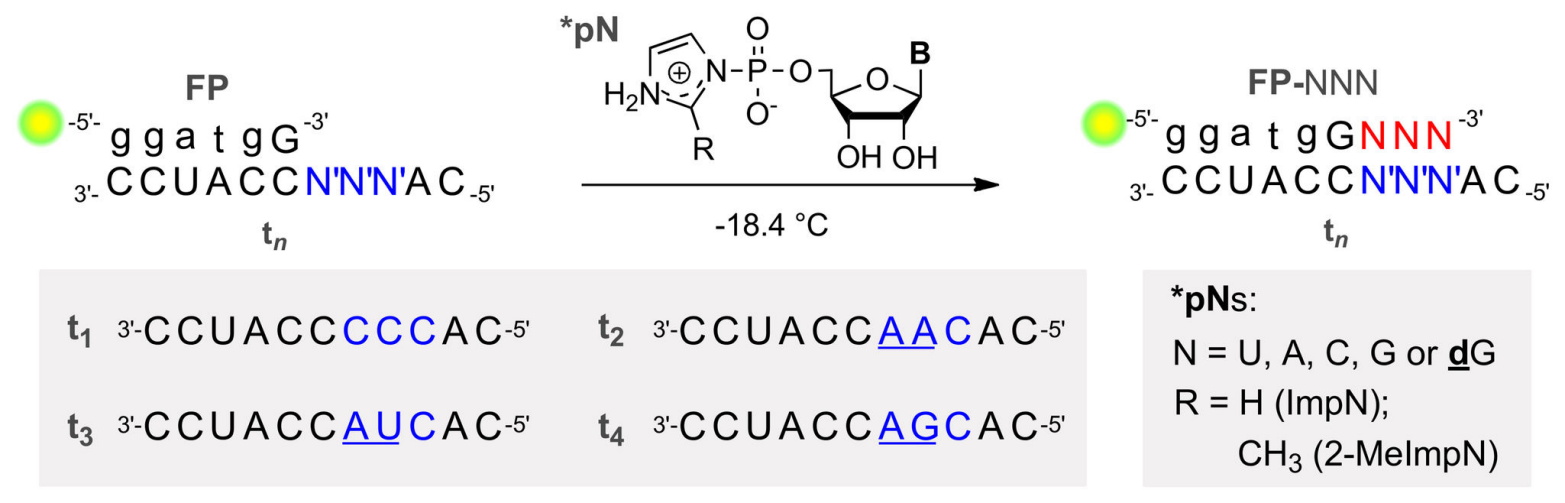
Experiment overview. 6-FAM fluorescently labeled primers (FP) bound to template **t**_**n**_ were incubated with mixtures of activated monomers (*pNs) at -18.4 °C. As emphasized in the schematic, the copying a motif of three template residues (blue) was investigated. The **t**_**1**_ motif is highly conducive to enzyme-free primer elongation, while the underlined motifs in **t**_**2**_-**t**_**4**_ are the sequences reported to block such reactions. Green spot = 6-FAM marker, small letters = deoxynucleotides (only in sequence drawings). B = nucleobase.

Our primary aim is to show that primer extension can occur across “blocking” sequence motifs when performed in the eutectic phase in ice. To this end, the criterion was to extend primers by three nucleosides when bound to templates presenting the AA, AU or AG motif (RNA templates). As it has been reported that poly(U) efficiently templates the synthesis of long oligo(A) sequences in eutectic ice [23] the motifs starting with a templating U (UA, UU) were omitted from this study. The initiation of the extension reaction is a critical step [24] and thus, due to a more favorable base-stacking interaction, insertion of purines is favored compared to pyrimidines. Hence, the elongation of a fluorescently labeled primer (FP) was studied in presence of templates containing a blocking motif, followed by a C-residue (N′N′C) (Figure 1). In order to accumulate triply extended primers (FP+3), typically, the reactions were terminated by insertion of a 2′-deoxyguanosine monomer (**d**G, instead of G). 2’-Deoxynucleotides prevents further elongation since their 3′-OH has a very low reactivity towards incoming 5′-phosphoramidates [25].

The secondary focus of this work is the assessment of sequence information transfer and regioselectivity during enzyme-free template copying in the eutectic phase. More precisely, to determine whether the triply extended primers contain a high fraction of template-directed product and which regioisomer of phosphodiester bonds predominates.

## Materials and Methods

### Monomer activation

Nucleotide 5′-imidazoyl monophosphates (ImpNs) and 5′-(2-methyl) imidazoyl monophosphates (2-MeImpNs) were produced from their respective free acid NMP’s (Sigma) and imidazole, using dithiodipyridine, triphenylphosphine and triethylamine in DMF (all Sigma, ACS grade). Synthesis and purification was performed according to a literature procedure [19,26].

### Primer extension experiments

A fluorescently labeled DNA-RNA hybrid oligomer with only one ribonucleotide at the 3′-end was used as primer throughout all experiments ^5^,′(6-FAM)-dGdGdA dTdGG^3^′, incubated with the specified templates and monomer mixtures (Figure 1). Stock solutions of activated monomers (*pNs), oligomers (Dharmacon or Thermohybaid; both Thermo, Fisher), morpholinoethylsulfonic acid (MES buffer), lead(II) nitrate, magnesium nitrate (all Sigma, p.a. grade) prepared in Millipore™ - MilliQ water. Reaction mixtures were prepared in 0.5 ml microtubes (a total volume of 100 µl). Sample composition: buffer (5 mM MES, pH 6.5), primer (**FP**, 10 µM), either one of the templates (**t_n_**, 20 µM), ImpNs or 2-MeImpNs (fresh 10X solutions, final concentrations: U, 1.75 mM; A, 0.75 mM; C,1.25 mM and G, 0.6 mM, if not otherwise indicated), 5.2 mM Mg(NO_3_)_2_, and 1.2 mM Pb(NO_3_)_2_.

The primer and template in buffer solution were first annealed (1 min at 90°C, left at 20 °C for 5 min), then placed in an ice-bath at 0 °C. Salts and monomers were added after the annealing and directly before incubation at -18.4°C for 5 or 14 days. The reported yields represent the total conversion of the primer calculated as the ratio between the sum of all newly appearing peak areas and the initial peak area of the primer at time t_0_.

### HPLC Analysis

The 6-carboxyfluoescein (6-FAM) labeled primer allowed fluorescence detection with linear quantification (i.e. no hypochromic effects) of sub-nanomolar amounts by HPLC [7]. In contrast to the electrophoresis technique previously used [4-6,15], anion-exchange HPLC columns specifically designed for nucleic acids are sensitive to sequence and regiochemistry of oligomers apart from separating by backbone length (charge) [27,28]. Solvents: NaClO_4_ (EMSURE^®^, Merck KGaA) and Trizma®-buffer (≥99.9%, Sigma) in MilliQ water. A: 5 mM Trizma, pH 8 or 9, B: 250mM NaClO_4_ in A.

All chromatograms were recorded using an Agilent 1200 system with diode-array UV detector (DAD) and fluorescence detector (FLD) using a Dionex^®^ DNAPac PA-200 column. Gradient: 0-2 min, 0% B; 2-42 min, convex gradient to 65% B, 42-45 min 80% B, fluorescence detection: λ_ex_ = 494 nm, λ_ex_ = 520 nm. Typical injections were 20 µl of an EDTA quenched aliquot of the reaction mixture (20 µl reaction mixture, 4 µl 0.1 M EDTA to bind divalent cations; diluted to 40 µl with H_2_O).

### Enzymatic assays of crude reaction mixtures

To aliquots of 20 µl of the reaction mixtures 1 µl RNAse ONE™ solution (10 u/µl), 3 µl 10X reaction buffer (PROMEGA) and 6 µl MilliQ water were added and incubated for 1 h at 37°C. RNAse ONE is an endonuclease, cleaving for 3′–5′-phosphodiester linkages specifically. The digestion products and an untreated control were analyzed as previously by HPLC, as the DNA-RNA hybrid primer and thus the fluorescent signal remain intact after RNAse treatment.

### Enzymatic assays of isolated products

Three identical reaction batches were pooled, lyophilized and resuspended in 100 µl MilliQ water. The entire volume was injected on HPLC and the relevant fractions collected (V_fraction_ ≤ 1 ml, contained 100-140 mM NaClO_4_). After precipitation with acetone (4 ml, -20 °C), the oligomer material was pelleted (0 °C, 4400 rpm, 30 min) and decanted, then washed with 1 ml acetone and pelleted again. The faint pellet was dried under N_2_ and resuspended in 100 µl H_2_O. A 20 µl aliquot was directly used for HPLC as a reference, while the remaining 80 µl incubated with RNAse ONE (2 µl, 10u/µl) and reaction buffer for 60 min at 37°C (total volume 100 µl). The entire mixture was then injected for HPLC. All quantitative data were normalized with respect to the area below the isolated peaks in the chromatogram obtained during the purification and thus reported as % of total isolated FP+3 products.

### Mass spectrometry

Mass spectra were obtained by Matrix Assisted Laser Desorption Ionization and Time of Flight detection (MALDI-TOF) using a Bruker Smartbeam III instrument (Bruker Daltonics, Bremen, Germany). The instrument was operated in linear positive ion mode at a detector gain factor of 7.7. Mass ranges were adjusted to target mass ± 1 nt (± >400m/z). Matrix: 300 mM trihydroxyacetophenone (THAP) in ethanol, containing 30% v/v 0.1 M diammonium citrate in water (both Sigma, p.a.). HPLC fractions (pH 9) were desalted prior to analysis using Empore® Extraction Disk material (purchased from 3M) in a 200 µl pipette tip. Wash: 100 µl 0.5% acetate in water; load: entire HPLC fraction; elute: 40 µl 0.5% acetate in 80% acetonitrile/water, v/v. Acetonitrile evaporated under a flow of N_2_ before applying 1 µl sample aliquots to 1 µl matrix droplets on the MALDI target.

## Results

Extension assays carried out in the presence of various RNA templates (**t_1_**-**t_4_**) individually (Figure 1) are presented in four separate sets of conditions, setups **1**-**4** (Table 1). All **FP** and **t_n_** form the same 6 bp duplex, with calculated melting temperatures (T_m_) above 25 °C [29] at the investigated Na^+^ and Mg^2+^ concentrations. The use of a hybrid primer (5 DNA, 1 RNA nt) allowed treatment with RNAse without digesting the primer itself, when investigating the regiochemistry of the monomer insertions. The primer-template association thus mostly has the character of a DNA-RNA duplex. Theoretically, it involves a departure from the strict C3′-endo conformation of the ribose in a pure RNA duplex (A-form helix), which has been proposed to be conducive to template-directed primer extensions [30]. However, it is questionable, whether a strong stabilization of any sugar puckering conformation can be invoked for such a short double stranded region (6 bp).

**Table 1:** Overview of primer extension setups.

**Setup**	**Template** (motif)	*pNs	**Primer conversion** (%)	**Yield of** FP+3 (%)^a^
**1**	**t_1_** (CCC)	G†	54 ± 3	32 ± 2^a^
	**t_2_** (AAC)	U†	47 ± 4	15 ± 4^a^
		U†, G†	45 ± 1	7 ± 0.2^a^
**2**	**t_2_** (AAC)	U, G	36^b^	7^a,b^
		U, G/dG (1:1)	47^c^	9^a,c^
		U, **d**G	44 ± 8	7 ± 3
**2a**	**t_2_** (AAC)	U, A, **d**G^c^	43^c^	4.5^c^
**3**	**t_3_** (AUC)	U, A, **d**G	38 ± 5	4 ± 1
**4**	**t_4_** (AGC)	U, C, **d**G	38 ± 4	3 ± 0.4

Yields obtained after 14 days reaction time with 2-MeImpNs or †: 5 days with ImpNs. Standard deviations given when available. ^a^ Yield includes longer products (i.e. FP+(3-n) when G was present, compared to when only **d**G was used and the extension efficiently stalled at **FP**-NN**d**G). ^b^control, only performed once. ^c^ average of two experiments.

Polymerization reactions were performed under previously optimized conditions, i.e. concentrations of activated monomers, metal ions and buffer conditions [14,15]. For each activated nucleotide a maximum of initial reaction rates was observed at a different concentration [15], which is the concentration used in the present work.

After incubation at -18.4 °C for up to 14 days in the presence of either of the templates (**t_n_**, see Figure 1), the products were characterized by HPLC in conjunction with mass spectrometry (MS) and RNAse assays.

Product sequences will be denoted, in the form **FP**- ^x^N^x^N (FP = fluorescent primer; regiochemistry indicated if applicable: x = 2′ or 3′ for a 2′-5′- or a 3′–5′-linkage, respectively; N = U, A, C, G or **d**G). Product residues that mismatch with the template are indicated by circumflexes, e.g., Û.

### Setup 1 – Control studies for template-directed primer extensions in the eutectic phase in ice

To assess the efficiency of primer extension in the current sequence design and under the chosen conditions, a set of experiments was performed in a non-competitive manner (i.e. only one monomer present) and subsequently in a competitive setup. Initial controls showed that the insertion of activated nucleotides strongly depended on the presence of metal ions, Mg^2+^ and Pb^2+^ (see Supporting Information, Figure S1 in File S1).

The total conversion of primer into elongated products was used as a basic measure of efficiency. In the presence of ImpG monomers (0.6 mM) and **t_1_** about 54% of the primer material was converted into elongated product. In comparison, the elongation with ImpU (1.75 mM) in presence of **t_2_** showed 47% conversion (Table 1).

The elongation with G in the presence of **t_1_** (G/**t_1_**) stalled after the incorporation a fourth, non-cognate nucleotide (**FP**-GGGĜ, Figure S13 in File S1) and the main product was **FP**-GGG. In case of U/**t_2_**, the template-specific product FP-UU had the highest abundance as well, however, longer products, up to FP-(U)_7_, were observed (Figure S12 in File S1). These observations indicated that the fidelity for insertion of guanosine alone was higher than that of uridine.

The elongation with a *pU/*pG mixture in presence of **t_2_** showed extension yields slightly below those observed under non-competitive conditions (Table 1). HPLC analysis highlighted a number of new product peaks in the range of FP+2 to FP+5 compared to the reaction with *pU alone. However, a notable misincorporation of G was also observed (Figure S2C in File S1).

The yields of primer that was extended by at least three monomers (FP+(3-*n*)) where expectedly highest for the G/**t**_1_ setup (32%). While in the U/**t_2_** setup still 15% of the primer was extended to FP+3 or longer, this figure was only about 7% in the competitive setup. These results clearly point to an effect of misincorporations on further extension steps, which will be discussed later.

In controls, where single monomers were incubated with the primer alone (non-templated reaction), still surprisingly high conversions where observed (29% and 54% for ImpG and ImpU, respectively). The effects of a lacking template-strand, however, become strongly evident, when measuring the yields of the longer products: only 2% of FP-(G)_3—n_ and 5% of of FP-(U)_3—n_ products were observed (data not shown). These results indicate a higher template dependence of guanosine insertions vs. uridine insertions in the eutectic phase in ice.

Note that the monomer concentrations used during competitive assays were not at equal stoichiometry, nor do they reflect the abundance of complementary template bases. In respect to earlier results, it has to be stressed that departure from the optimal ImpN concentrations by ±0.5 mM results in some cases in lowering the reaction rates by up to a factor of two [15]. Thus, when performing the enzyme-free primer extensions with competing monomers, we argue that supplying each *pN at the individual optimal concentration, where their respective insertion rates were observed to be the highest [15], provides a superior balance in insertion frequencies compared to supplying each *pNs at the same concentration. In a prebiotic scenario, neither optimal concentrations nor equal stoichiometry could possibly be expected. Monomer concentrations would rather depend on the efficiency of their prebiotic syntheses. Consequently, in this work it was merely of interest to study the most efficient system, which, however, is dependent on absolute concentrations rather than a certain ratio between monomers.

Illustrating this statement, preliminary studies established, that using a 1:1 stoichiometry of ImpU and ImpG at each 0.875 mM ([ImpN] = 1.75 mM, optimal concentration for ImpU) the relative (mis-) incorporation of G was lower, than when using each at optimal concentrations (Figure S2B in File S1).

Hence, during competitive reactions, the total concentration of monomers was higher with respect to the individual assays. However, the highest total monomer concentration in this study was 3.6 mM, staying well below a total monomer concentration of 5 mM, where a significant lowering in the overall reaction yields was observed (Figure S2D in File S1). At 5 mM the resulting liquid volume and the structure of the eutectic phase were likely affected (as it depends on the starting concentration of all solutes), thereby lowering the overall reaction efficiency.

### Setup 2 – the AAC sequence motif

For the following experiments, 5′-phosphoro(2-methyl) imidazolide nucleosides (2-MeImpNs) were used instead of the imidazolide variants (ImpNs), to facilitate the comparison with the various works by Orgel and co-workers [4-6,31-33]. Their results had demonstrated that 2-MeImpG outperformed all other imidazole variants during polymerization in the presence of poly(C). That is, they produced longer oligomers and showed a higher regioselectivity towards 3′–5′-linkages [24]. In the eutectic phase, however, the Pb^2+^/Mg^2+^ catalyzed self-condensation of ImpU, was shown to provide slightly higher amounts of oligouridylates, than 2-MeImpU [13].

2-methyl imidazole derivatives are slightly less reactive but also less prone to hydrolysis than their imidazole counterparts [34]. Thus, they can be incubated in an active form for longer times -14 instead of 5 days. As ImpNs were virtually spent after 5 days, incubation of 14 days did not result in higher yields in those experiments (data not shown).

The yields of the competitive elongation in presence of **t_2,_** (∙ ∙ ∙**AAC**AC^5^′, *pU/*p**d**G) were somewhat lowered by the change of activation group (45% and 36% for ImpNs and 2-MeImpNs, respectively, Table 1), even though the latter samples were incubated 14 days.

**Figure 2:**
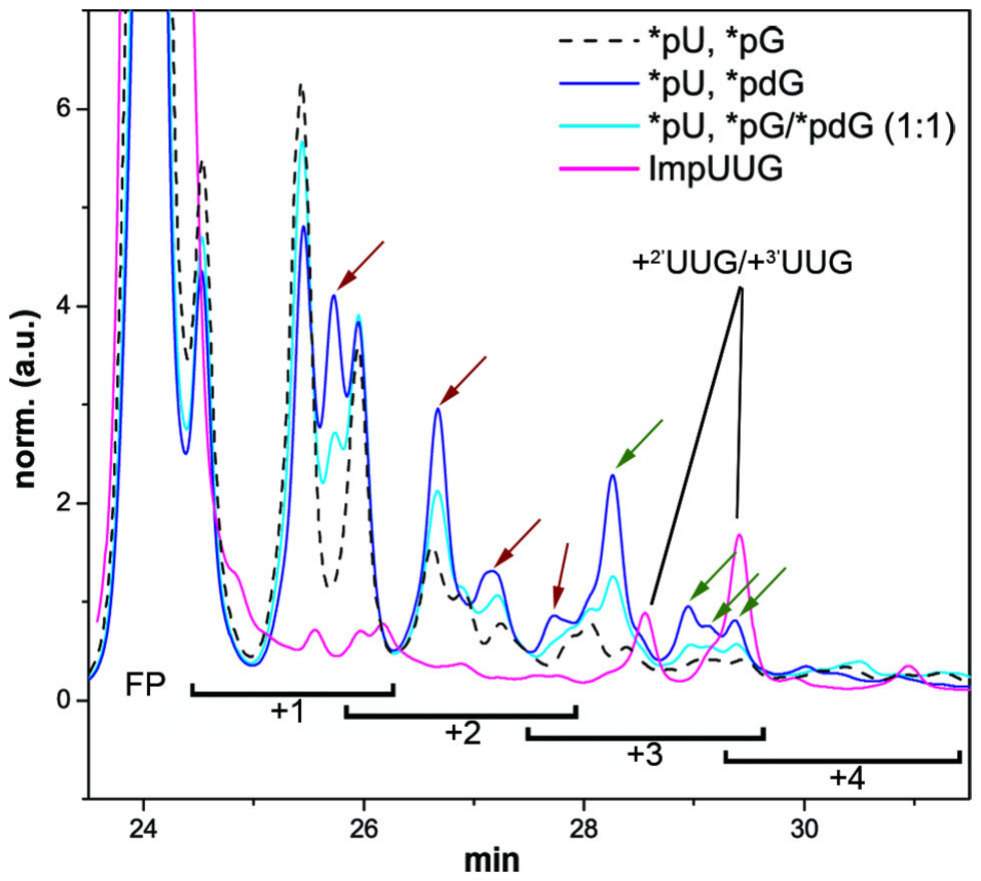
Product accumulation after dG incorporation. HPLC chromatograms of setup **2** reaction mixtures after 14 days incubation at -18.4°C: black stipples, *pU, *pG; cyan, *pU, *pG/*pdG (1:1); blue, *pU, *pdG. Red: ligation of ImpUUG as internal control for the elution range of FP+3 products (using 2.5 µM **FP** and 7.5 µM ImpUUG, see Supporting Materials and Methods in File S1 for synthesis of ImpUUG). Arrows indicate accumulated products due to dG insertions; red: non-specific insertion, green: template-directed ones.

To prevent further extension of FP+3 products observed in setup **1**, **d**G monomers were introduced instead of G. The accumulation of products containing **d**G was monitored by carrying out the reaction with *pG, *pG/*p**d**G (1:1) or *p**d**G. The accumulation of certain peaks is illustrated in Figure 2, occurring both in the +1, +2 and +3 region of the chromatogram.

While the increase in overall primer conversion (approx. 44%) in the *pdG reaction resulted in part from the misincorporations at +1 and +2 positions, the yield of FP+3 products contained no bias from these misincorporations. Only minute amounts of primer extended by four or more nucleotides (FP+(4-*n*)) were detected, a fact that confirms the efficient inhibition of further elongation by dG, due to the lack of the 2′-OH group. Thus, a misincorporation of dG at +1 or +2 position effectively decreases the number of strands that reach FP+3. Using **d**G, doubly extended primers (FP+2) were efficiently capped by the insertion of **d**G across C as the third extension, thus allowing the accumulation of FP-UU**d**G products. The yields of **FP**+NN**d**G were thus approximately equal to those of all FP+(3-*n*) products in experiments without **d**G (see Table 1 footnote). Conversely, in reactions with *pG, FP+3 products where further converted into FP+(4-*n*) strands, which rendered the measurement of yields less reliable due to increasingly difficult chromatographic separation down to baseline level. In fact, a noteworthy uncertainty in placement of an integration baseline arises due the multitude of peaks that results from the various regioisomers alone (2^*n*^ possibilities for FP+*n*). Hence, linear (flat) baselines were carefully placed for the analysis of the chromatograms, to counteract this uncertainty as much as possible.

**Figure 3:**
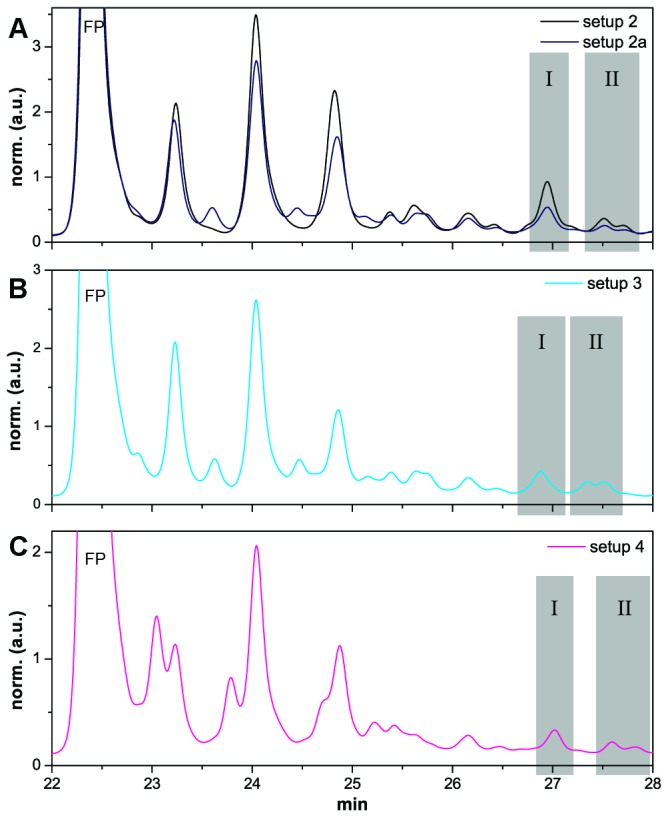
Overlay of HPLC traces of setups 2–4. (**A**) Setup **2**, 2-MeImpU, -dG; Setup **2a** -2-MeImpU, -A, -dG. (**B**) Setup **3**, 2-MeImpU, -A, -dG. (**C**) Setup 5, 2-MeImpU, -C, -dG. Grey boxes indicate FP+3 product fractions analyzed by MALDI-TOF MS.

**Figure 4:**
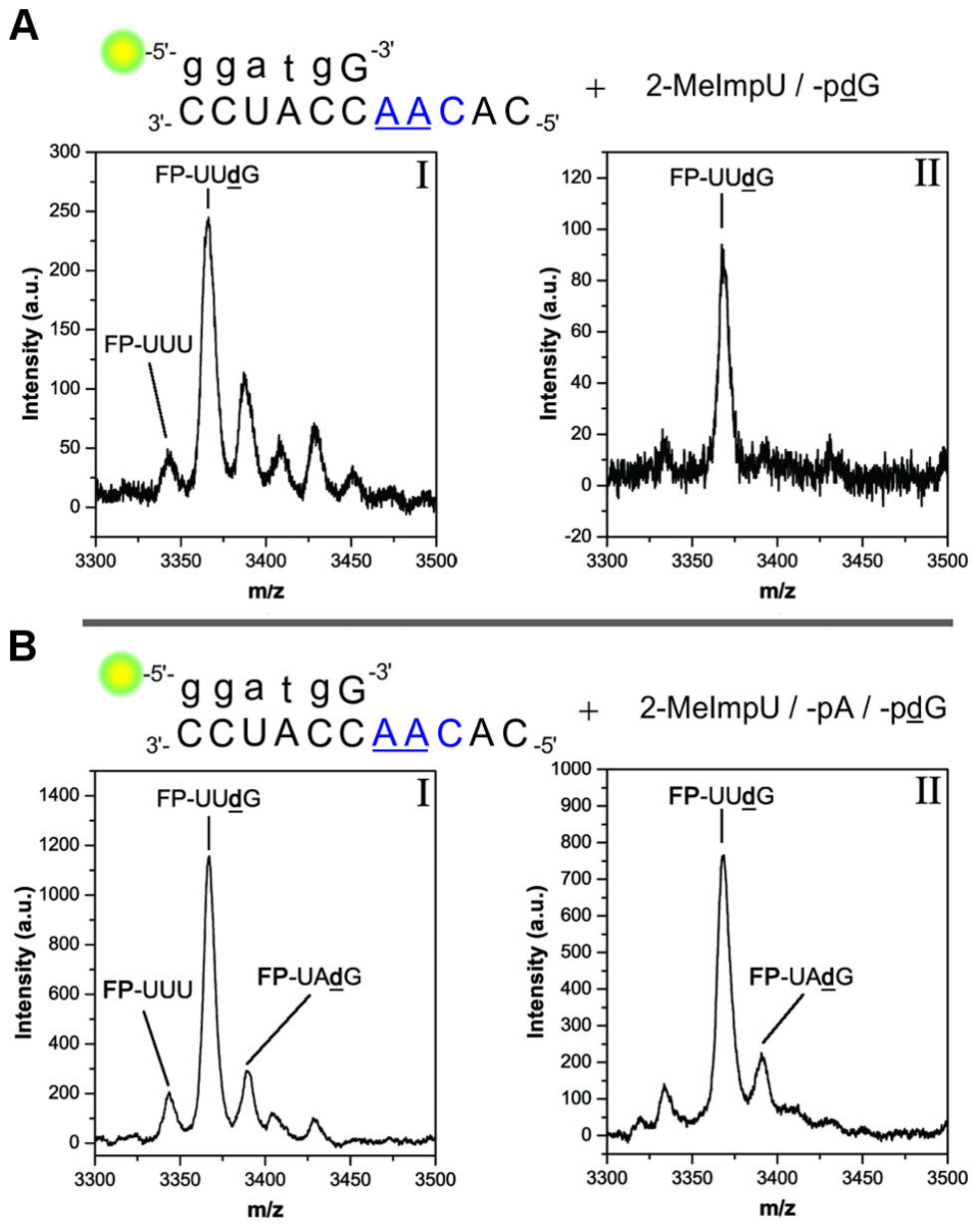
Analysis of FP+3 products in setup 2 and 2a. Oligomers identified by MALDI-TOF MS (**A**) Setup **2** – (I): FP-UUÛ (3344.26 g/mol, found m/z 3343.46, trace); FP-UUdG (3367.26 g/mol, found m/z 3365.86, and Na^+^-adducts) (II). FP-UUdG (3367.26 g/mol, found m/z 3367.63). (**B**) Setup **2a** – (I): FP-UUÛ (3344.26 g/mol, found m/z = 3343.46, trace); FP-UUdG (3367.26 g/mol, found m/z 3365.86), FP-UÂdG (3390.33 g/mol, found m/z 3389.08) (II). FP-UUdG (3367.26 g/mol, found m/z 3367.63); FP-UÂdG (3390.33 g/mol, found m/z 3390.66).

In setup **2a**, the non-cognate *pA was added as a third monomer, and the HPLC traces were compared with those of setup **2**. The differences in the traces were only minor misincorporations of A monomers at +1 and +2 positions in setup **2a**, while FP+3 peaks seemed almost identical (Figure 3A). In the mass spectra obtained from the isolated FP+3 fractions I and II (as marked in Figure 3A) **FP**-UU**d**G was observed as the main product in both setups (Figure 4). A trace amount of the template unspecific **FP**-UÂ**d**G was also observed in setup **2a** (Figure 4B). MS analysis of all shorter products for setup **2** are reported in the supporting information (Table S1, Figures S3 and S4 in File S1).

Even though A and **d**G residues are not distinguishable by mass, the sequence combinations not ending on **d**G were likely not formed, as an accumulation of the +3 peak was also observed with the additional *pA present in setup **2a**. Thus, sequence combinations such as **FP**-[U_1_A_2_] or **FP**-[U_2_A_1_] ([N_n_N_m_] = sequence combinations, individual abundances in subscript) were unlikely to have formed. The FP+3 yield for setup **2a** was lower (4.5%) than for setup **2** (7%). This observation could be explained by the formation of **FP**-Â and subsequent stalling caused by the mismatch [25,35], which reduced the primer material that could be further elongated. Altogether, the results indicated a strongly template-directed character of the triply elongated primers.

### Setup 3 – the AUC sequence motif

**Figure 5:**
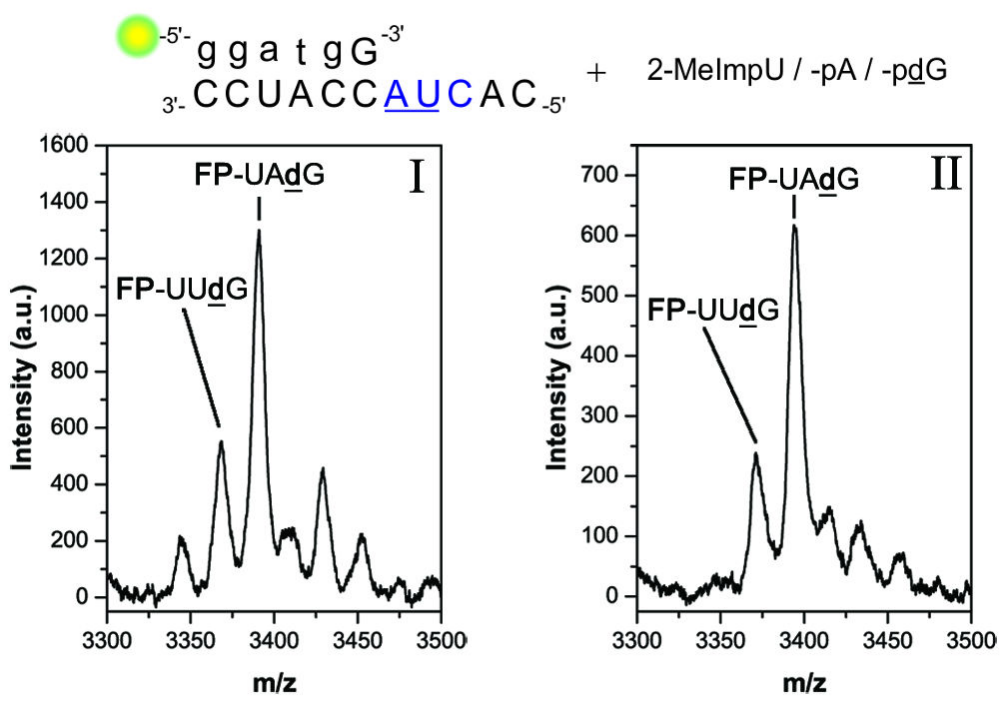
MALDI-TOF MS of FP+3 products in setup 3. Identified oligomers: FP-UAdG (3390.33 g/mol, found m/z: 3390.77 (I) and 3390.56 (II), FP-UUdG (3367.26 g/mol, found m/z: 3368.30 (I) and 3367.40 (II). FP-UUU (3344.26 g/mol, found m/z: 3343.46, trace in (I)).

By carrying out the primer elongation in the presence of **t_3_**, evidence for a faithful copying of the **AUC** motif was investigated. HPLC analysis showed the accumulation of triply elongated primer (FP+3), as previously described for setup **2**, reaching approximately 4% yield of total primer material. The HPLC trace of the FP+3 products, showed a similar pattern of peaks, however, eluting at slightly earlier times (Figure 3B). The isolated fractions I and II of these products were analyzed by MALDI-TOF MS (Figure 5). The results indicated that the main product formed was **FP**-UA**d**G, but that a fraction of **FP**-UÛ**d**G was also present. MS analysis of FP+1 and FP+2 products showed, that only trace amounts of **FP**-Â mismatch side-products (observed in the HPLC chromatogram) were converted into **FP**-Â**d**Ĝ or **FP-**ÂA (Table S2, Figures S5 and S6 in File S1), another example of elongation stalling after misincorporation.

In a control reaction without *p**d**G, the lack of accumulation of FP+3 products confirmed a preferential insertion of G across C in the complete system. In another control, where *pU monomers were omitted, only traces of FP+3 products were found. These and other controls are displayed in Table S4 and Figure S9 in File S1.

Combining the results from MS analysis and the control reactions, the production of the following sequences was shown to be unlikely: **FP**-[U_2_A_1_] (same m/z as **FP**-UU**d**G), **FP**-[U_1_A_2_] and **FP**-ÂÛ**d**G (same m/z as **FP**-UA**d**G). Furthermore, no products with masses corresponding to **FP**-ÂA**d**G or **FP**-ÂAÂ were observed. Altogether, the data led us to conclude that the main portion of triply elongated primer was indeed the template specific product **FP**-UA**d**G.

### Setup 4 – the AGC sequence motif

**Figure 6:**
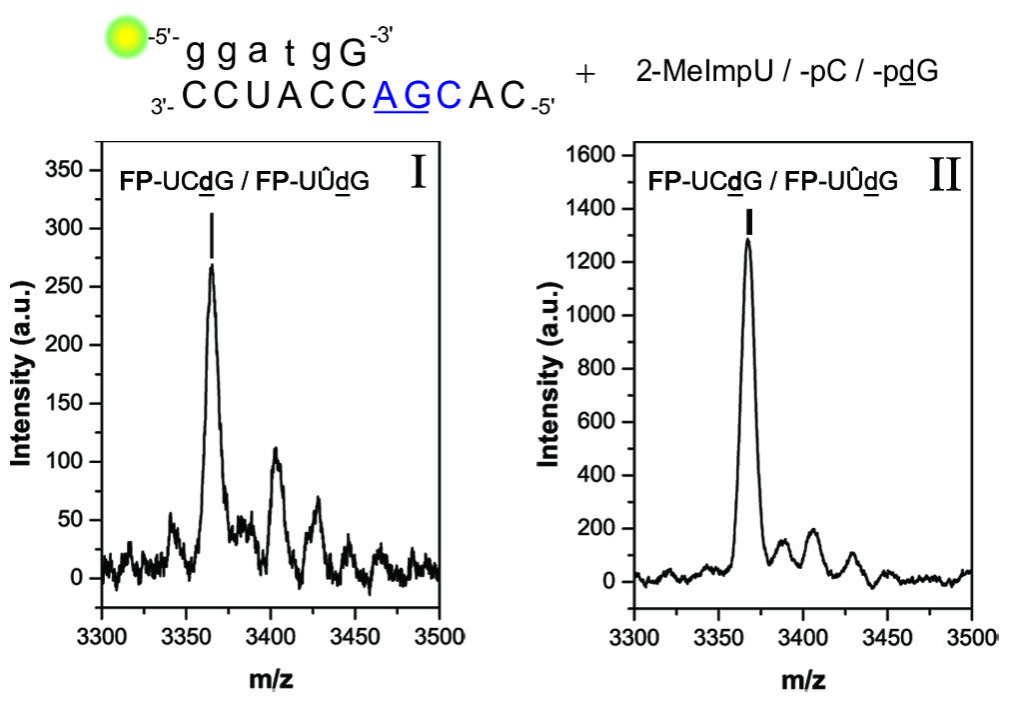
MALDI-TOF MS of FP+3 products in setup 4. Main m/z abundance in both (I) and (II) correspond to FP-UCdG (3366.28 g/mol) well as FP-UUdG (3366.28 g/mol) within the resolution of the measurement. Found (I) m/z 3365.36; (II) found m/z 3367.43.

The extension of **FP** in presence of **t_4_** formed FP+3 products in 3% yield, using a mixture of 2-MeImpU, -C and -**d**G (see Table 1). Even though, the second canonical elongation in this experiment is promoted by a C·G base pair, the yield of triply elongated primer was the lowest of all setups. This result could be in part attributed to the significant misincorporation of C in the FP+1 position (in addition to **FP**-**d**Ĝ) and subsequent mismatch stalling (Figure 3C). The isolated FP+3 product fractions I and II likely contained both **FP**-UC**d**G and **FP**-UÛ**d**G as their masses could not be resolved by MALDI-TOF MS (Δm/z = 0.98 for U vs. C) (Figure 6). During HPLC analysis, the triply elongated primers from setup **4** reactions eluted slightly later than those of setup **2**, i.e. the main products formed in setup **4** do not superimpose with the products from setup **2** (i.e. **FP**-UU**d**G). Nonetheless, **FP**-UÛ**d**G was produced in a control experiment carried out in absence of *pC with **t_4_** (Figure S10 in File S1). This result could be explained by the formation of wobble pairing (U·G), and by the low template specificity observed during non-competitive conditions for U monomers in the eutectic phase.

Other possible sequences, **FP**-ĈC**d**G and **FP**-ĈÛ**d**G were less likely to have formed in significant amounts due to their mismatch in the FP+1 position. Indeed, control experiments where **FP** and **t_4_** were incubated with *pC and *p**d**G only, showed only traces of FP+3 products (not detectable by MS). Conversely, starting the extension from a pre-synthesized **FP**-^3^′U primer with monomers *pC and *p**d**G efficiently produced **FP**-UC**d**G (for all controls, see Table S4 and Figure S10 in File S1). Thus, it can be argued that only if FP-U is formed, further elongation is efficient.

In combination, the findings from MS analysis and the control reactions indicated that both the template-specific sequence **FP**-UC**d**G and the mismatch sequence **FP**-UÛ**d**G were formed in setup **4**. MALDI-TOF MS data of all products that could be isolated from HPLC in sufficient quantities are reported in the supplementary information (Table S3, Figures S7 and S8 in File S1).

### Regiochemistry of extension products

Enzymatic degradation assays with the 3′–5′-specific RNAse ONE were performed for each setup. The amount of primer with a 3′-terminal phosphate (**FP**-p) formed during digestion reflects the relative amount of 3′–5′-linkages in the +1 extension step compared to an untreated control (Figure S11 in File S1). The resulting ratios of 2′-5′- to 3′–5′-linkages are reported in Table 2. HPLC analysis of setup **1** digestion products also facilitated the evaluation of the synthesized strand lengths (Figures S12 and S13 in File S1).

**Table 2:** Regioselectivity of the first insertion in total elongated primer material.

**Setup**	**Monomers**	**Ratio of 2′-5′ vs. 3′–5′**
1	ImpG	0.8 : 1
	ImpU	2.5 : 1
	ImpU, -G	1.6 : 1
2	2-MeImpU, -**d**G	1.7 : 1
3	2-MeImpU, -A, -**d**G	1.4 : 1
4	2-MeImpU, -C, -**d**G	0.9 : 1

For reactions with monomer mixtures, apparent ratios are reported as they could not be deconstructed into the tendencies for each monomer. Nonetheless, in presence of U, 2′-5′-linkages were favored (setup **2**). Taking into account the insertions of A in setup **3**, the excess of 2′-5′ linkages was less pronounced. Surprisingly, in setup **4** 3′–5′-linkages were slightly favored, a fact that was attributed to the presence of C monomers. On closer inspection of the FP+3 region of the product chromatograms after treatment with RNAse ONE (Figure S14 in File S1) it was established, that the first peak (i.e. fraction I) was resistant to digestion in setups **2** and **4**, and partly resistant in setup **3**. The later eluting peaks (i.e., fraction II) on the other hand were almost completely digested in all setups. In turn, the product fractions I and II (i.e. FP+3 products) for each setup were then assayed separately in order to gather information about the regioselectivity of the triply elongated products.

From a quantitative analysis of degradation products, the relative abundances of their pre-treatment educts were established (Table 3). Generally, these data substantiated that for each setup the fractions I contained more 2′-5′-linked material than the fractions II. In case of setup **2**, taking into account, the higher yield in fraction I, one obtains a regioselectivity of about 2:1 in favor of 2′-5′-linkages. In setups **3** and **4**, no strong preference was observed, although in setup **3**, **FP**-^3^′N^x^N^x^N regioisomers were more abundant than **FP**-^2^′N^x^N^x^N variants. Interestingly, the group of regioisomers starting with a 2′-5′-linkage contained a high relative amount of **FP-** ^2^′N^2′^N^2′^N in all setups, indicating that 2′-5′-linkages might direct towards further 2′-5′-linkages downstream. Of total FP+3 only 5-11% **FP** ^2^′-N^3^′-N^x^-N and 1-5% **FP** ^2^′-N^2^′-N^3^′-N were formed for all setups (see Table S5 in File S1 for details of mixed 2′/3′ product abundances).

**Table 3:** Regiochemistry of triply elongated primers.

**Setup**	**Fraction**	**Relative abundances (to total FP+3 product**)** of** ^a^	
(template, monomers)		**FP**-^3′^N^x^N^x^N (%)	**FP**-^2′^N^x^N^x^N^b^ (%)
**2** (t_2_, U/dG)	I	8	60
	II	24	8
	**total**	**32**	**68**
**3** (t_3_, U/A/**d**G)	I	22	28
	II	35	15
	**total**	**57**	**43**
**4** (t_4_, U/C/**d**G)	I	19	46
	II	28	7
	**total**	**47**	**53**

^a^ 2′ = 2′-5′-, 3′ = 3′- 5′-linkage, x = both regioisomers possible. ^b^ for a more detailed analysis see Table S5 in File S1: breakdown into **FP**-^2′^ N^3′^N^x^N, FP-^2′^ N^2′^N^3′^N and RNAse resistant **FP**-^2′^ N^2′^N^2′^N.

In each setup, a large portion of FP+3 material was resistant to the enzyme, especially the main peak of fraction I for setups **2** and **4** (Figure S15 in File S1). While control digestions of a synthetic standards of the FP+3 sequences showed complete digestion (data not shown), the percentage of undigested material was 58%, 29% and 46% for setups **2**, **3** and **4**, respectively (Table S5 in File S1). Surprisingly, setup **3** showed the least resistant product material of all setups, despite an excess of 2′-5′-linkages in the first insertion (Table 2). For setup **4**, the comparably higher propensity to form a 3′–5′-bond during the first insertion did not significantly lower the relative amount of RNAse resistant FP+3 product.

The enzymatic assay of FP+3 material also provided additional information about the formed sequences, which could be inferred from the retention times of the FP-N-p peaks (**FP-** ^2^′N^3′^N^x^N → FP**-** ^2^′N-p) of the digested material. An overlay of digestion products from the fraction I and II samples of setup **3** and **4** confirmed the presence of **FP**-^2^′U-p but also showed some **FP**-^2^′Â-p and **FP**-^2^′Ĉ-p digestion product. Thus, traces of FP-ÂÛ**d**G and of **FP**-ĈĈ**d**G/**FP**-ĈÛ**d**G were formed in setups **3** and **4**, respectively (Figure S16 in File S1).

## Discussion

### Effectiveness of primer-elongations in the eutectic phase

As listed in Table 1, the overall conversion of primer into elongated products, ranges from 36% to 47% with all templates containing adenine residues. The “benchmark” with the CCC motif and ImpG monomers did not show primer conversions above 60%, while the primer material in corresponding elongation experiments in aqueous phase usually consume all initial primer material [11]. This fact might be attributed to the environment conditions in the eutectic phase, where a certain de-mixing of the reactants in a semi-compartmentalized system cannot be ruled out.

In fact, very little is known about the surface effects and spatial constraints on nucleic acid oligomers within the brine channels in the eutectic reaction environment. In conjunction with the successful template copying using an artificial ribozyme in the eutectic water-ice phase, Holliger and co-workers [36] performed diffusion studies and freeze-fracture SEM. It was reported that in the eutectic phase, diffusivity was lowered by over three orders of magnitude, and the brine channels in the frozen samples became increasingly fragmented if the reaction mixtures were diluted prior to incubation.

Related to the present work, primer extension experiments were performed at -20 °C by Richert and co-workers (16) using 1-hydroxy-7-azabenzotriazole (HOAt) activated ribothymidine. For a reaction using a template with the AAC motif across the first three insertions 22% primer conversion after 7 days, and 36% after monomer replenishment and an incubation of 15 days were reported. In the present experiments, these conversion yields could be significantly increased in a one-pot reaction (~47% after 5 days using ImpU). Still, these competitive elongations do not match the >90% conversion reported, using short oligomers that bind downstream of the first open template residue, thereby supporting the elongation and restricting each copying step to a single monomer insertion [16].

In our study, systems that involved more than two nucleotides showed a slightly lower total conversion, and this effect was increasingly pronounced for the FP+3 product yield. Hey et al. reported such an effect, during competitive primer elongations which was termed cross-inhibition [11], which seemed to be dependent on the primer, as well as the template sequences. Arguably, this phenomenon could be explained by the effect of misincorporations on the reaction kinetics studied in by Chen and co-workers [25,35]. In their work, reaction rates were lowered by more than two orders of magnitude, when primer and template were mismatched at the elongation site. Thus, reaction setups that have a high propensity for misincorporations, e.g., due to wobble base-pairing or strong base stacking, would result in a strong stalling effect. In the present work, this stalling effect was apparent the control reactions with **t_3_** and **t_4_** where *pU was omitted, forcing a misincorporation in the first copying step. In the cases where *pU was present and *pC/*pA was omitted, however, the stalling was not as pronounced. The first insertion next to the primer would therefore significantly influence the extension yield or its stalling. This observation could be related to the mechanism of template-direction during primer extension, i.e., whether monomers pre-align on the template prior to forming backbone linkages or if extension occurs step-wise due to rapid exchange of monomers. The mechanistic aspects of chemical template-copying remain open questions to date.

In an assay where monomers compete for insertion over “reported blocking sequences” [6], misincorporations are likely to occur. Generally, the template specificity in the present work leaves room for optimization. All setups showed substantial misincorporations of G/dG in the first insertion and some template unspecific elongations with U (traces of FP-U_3_ and FP-U_4_). Focusing on FP+3 products in setups **2a** and **3**, both using U, A and **d**G in the presence of **t_2_** and **t_3_**, respectively, misincorporations were only leading to minor byproducts. In presence of the AA-motif template in setup **2a**, FP-UU**d**G was in clear excess of the unspecific product FP-UA**d**G, and vice versa for the AU-containing template, setup **3**. In setup **4**, the FP+3 products likely contained a mixture of **FP**-UC**d**G and **FP**-UÛ**d**G. As those products could neither be completely separated by HPLC nor distinguished by mass, their relative quantification remained elusive.

Nonetheless, control experiments showed that an elongation with a cognate monomer, favored the odds for further insertions. When pre-synthesized FP-U was elongated in the presence of **t_3_** and *pA/*p**d**G or **t_4_** and *pC/*p**d**G, a high conversion into FP+3 products was observed. Thus, provided that U is inserted across the A-residue, **FP**-UA**d**G and **FP**-UC**d**G would readily form (Figures S9D and S10D in File S1). Furthermore, controls, where one of the monomers was absent, showed that **FP**-ĈC**d**G (**t_3_**) and **FP**-ÂA**d**G (**t_4_**) were unlikely to form in setup **3** and **4** in the absence of *pU. By constrast, **FP**-UÛ**d**G was synthesized in notable amounts in setup **3** in the absence of *pA and in setup **4** in the absence of *pC, respectively. Table 4 summarizes the observed extension products in setups **2**-**4**.

**Table 4:** Overview of the elongation products detected by MALDI-TOF (ordered by abundance).

**Setup**	**Monomers**	**FP+3 main products**	**Side products**
**2**	U, **d**G	**FP**-UU**d**G	**FP**-UUÛ (tr.)
			**FP**-UUÛÛ (tr.)
**2a**	U, A, **d**G	**FP**-UU**d**G	**FP**-UÂ**d**G
			**FP**-UUÛ (tr)
**3**	U, A, **d**G	**FP**-UA**d**G	**FP**-UÛ**d**G
			**FP**-UUÛ (tr.)
**4**	U, C, **d**G	**FP**-UC**d**G	**FP**-ĈC**d**G/**FP**-ĈÛ**d**G
		**FP**-UÛ**d**G	**FP**-UUÛ (tr.)

tr. = trace.

In the work of Wu & Orgel [4-6], where ‘ACCCC’ and ‘TCCCC’ templates were successfully copied, only indirect evidence for the incorporation of U and A, respectively, could be provided. Exact control of product sequences, has only recently been shown by Richert and co-workers [17]. However, it was only possible through a surface-bound, step-wise procedure restricted by downstream binding strands. While their elegant approach is a benchmark for technological applications, answers to the open questions of the origin of information transfer need to be sought in unrestricted systems, and under competitive conditions.

For a better characterization of complex product mixtures in such reactions, sensitive analysis tools become of great importance. Recent reports of controlled partial digestion by immobilized Snake Venom Phosphodiesterase (3′→5′ exonuclease) promise new analysis possibilities for sub-nanomolar quantities of nucleic acid oligomers by MS sequencing or HPLC [37]. A polymerase-free sequencing method such as this, is necessary would overcome the problems with using, e.g., Next Generation Sequencing, from a substrate pool with mixed 2′-5′- and 3′–5′-backbone chemistry. To attain a more precise qualitative picture by mass spectrometry, a subtle way of creating a greater Δm/z between U and C residues, could be the use of non-canonical ribothymidine or uridine derivatives, e.g. 5-(3-aminopropyl) uridine that was shown to be a good substrate for T7 RNA polymerase [38]. In future experiments, the influence of the base-stacking between the nucleobase at the 3′-terminus of the primer and the incoming nucleotide should also be investigated, i.e. studying primers that end with an A, C or U.

### Regioselectivity in structured media

Wu & Orgel showed a high selectivity for 3′–5′-linkages in the copying of ‘ACCCC’ and ‘TCCCC’ DNA template motifs with 2-MeImpNs. This selectivity inverted towards 2′-5′-linkages if only one of the cognate monomers was supplied. The general trends in the present work indicated that ImpG slightly favors 3′–5′-linkages, whereas ImpU predominantly forms 2′-5′-linkages (Tables 2 and 3). Analysis of FP+3 products showed that a 2′-5′-linkage in the +1 or +2 position was rarely followed by a 3′–5′-linkage. Together with the observed enrichment in RNAse resistant FP+3 product (29-58%, vs. 12.5% by statistics, i.e. 1 of 2^3^ combinations), this fact might imply an inclination towards 2′-5′-homo-regioisomers. Conversely, it remained unclear whether this also applies for 3′–5′-linkages, potentially causing a divergence towards either homo-regioisomer. Such a change in backbone-geometry, might cause a repositioning of the nucleobase and affecting possible stacking interactions. Since 2′-5′ bonds are known to lower duplex melting temperatures, it could also be argued that the reaction attains a lesser template-directed character, as observed for U.

The regioselectivity of RNA polymerization can be strongly influenced by the choice of activation group. 1-methyladenine activated monomers in the presence of montmorillonite clay formed long oligomers (up to 50-mers) of 3′–5′-selective oligoadenylates and oligouridylates [39,40]. Such an activation group could help increasing regioselectivity towards 3′–5′-phosphodiesters in future experiments.

### Prospects for RNA replication

Since our results showed that AA, AU or AG template motifs did not block enzyme-free primer extensions, the eutectic phase in ice poses a promising reaction medium. Using freeze-and-thaw cycles [23] during the incubation time might improve the efficiency and also the fidelity of these reactions. Procedures or chemistries that make possible the replenishment of activated nucleotides are be necessary to achieve very high conversions, as shown by Richert and co-workers [17]. As mismatch stalling also takes effect in eutectic ice, a high template fidelity seems to be achievable, albeit at the cost of yields. If conversion rates are optimized, considerable yields of faithfully copied RNA strands might be obtained from a one-pot setup.

Further studies should focus on optimizing analysis tools and exploring reaction compositions of enzyme-free primer extension in ice. The stoichiometry between different monomers is likely to influence the extension yields and its sequence fidelity. Apart from the free energy of π-π-stacking and hydrogen bonding between adjacent and opposing nucleobases, respectively, other phenomena influence the reaction in the eutectic phase. Evidently, since a successful primer elongation is most efficient within a given range of monomer concentration, ice-surface interactions, solubility limits and other possible factors need to be investigated.

### An RNA world in ice

It may be surprising to propose the existence of an RNA world in ice during the Origin of Life on Earth or, for the matter, right now on the moons of planets in the solar system (e.g., Europa). The prebiotic plausibility of conditions conducive to the formation of eutectic phases in water/ice seems unlikely in the prevalent view of a hot early Earth. However, recent studies increasingly question this scenario and propose a colder history of the early Earth, consistent with the presence of ice deposits during the Hadean period and the transition into the Archaean. Indeed, taking into account the lower intensity of the young sun (approximately -30% 4 Gy ago) [41], the surface temperature would greatly depend on the rate of removal of CO_2_ by subduction of the oceanic crust [42]. Such subduction is linked to a rapid formation of continental crust, such as proposed by the Armstrong model from 1981 [43] and possibly substantiated by findings >4 Gy old zircons from the Acasta Gneiss formation in Northern Canada [44]. On the basis of these studies, Arndt and Nisbet even suggest that, not long after the Moon-forming impact, the early Earth might have cooled sufficiently to present a deep ice-covered ocean surface above flowing lava and submerged continental nuclei [45]. In the period from ~4.4 Gy until the end of the late heavy bombardment (3.8 Gy ago), the presence of liquid water would have been ensured by volcanic activity [45] while the frozen surface might have been periodically disrupted by foreign body impacts [46]. Due to the lack of extensive direct records, the investigation of refractory zirconium-containing pellets (formed 4.5-4.0 Gy ago) [47] has however led to contradicting theoretical interpretations. Nonetheless, on a cold earth, the conditions required to form a water-ice phase like the one used in this study would have certainly been fulfilled.

## Conclusion

The results of the present study demonstrate that the eutectic phase in water-ice is a potent environment for studying enzyme-free RNA replication, enabling the use of the full spectrum of canonical RNA nucleobases and possibly also modified ones. An important result of this work is that the competitive elongations of a fluorescently labeled primer was not blocked by the template motifs AA, AU or AG. These “blocks” could be passed through the insertion of three nucleotides. Triply elongated products could be accumulated by the insertion of **d**G across C as the third template residue. In particular, high relative amounts of template specific product were formed across AAC and AUC. During the copying of the AGC motif +3 products were formed as well and highest overall primer conversion was obtained in presence of all three cognate nucleotides. However, the exact product distribution (+UC**d**G, +UU**d**G, small amounts of +CC**d**G or +CU**d**G) remained elusive. The results indicate that the discrimination in favor of the cognate nucleobase by the template [15] previously observed in a one-nucleotide setup also applies to competitive reaction setups. Furthermore, it could be confirmed that the stalling of the elongation reaction after misincorporations [25], also occurs in the eutectic phase in ice.

These results augur well for the investigation of the origin of the replication in an RNA world and for potential technological and medical applications provided it allows for the copying of nucleic acids with a different backbone, or the unrestricted employment of modified nucleotides.

## Supporting Information

File S1 Combined Supporting Information document containing Supporting Materials and Methods, Tables S1-S5 and Figures S1-S16.
(PDF)

## References

[1] Woese CR (1967) The genetic code; the molecular basis for genetic expression. V iii. New York: Harper & Row . 200pp

[2] Gilbert W (1986) Origin of Life - the RNA World. Nature 319: 618-618. doi:10.1038/319618b0.

[3] Röthlingshöfer M, Kervio E, Lommel T, Plutowski U, Hochgesand A et al. (2008) Chemical primer extension in seconds. Angew Chem Int Ed 47: 6065-6068 doi:10.1002/anie.200801260. PubMed: 18613189. 10.1002/anie.200801260 18613189

[4] Wu T, Orgel LE (1992) Nonenzymatic Template-Directed Dynthesis on Oligodeoxycytidylate Sequences in Hairpin Oligonucleotides. J Am Chem Soc 114: 317-322. doi:10.1021/ja00027a040. PubMed: 11540927. 11540927 10.1021/ja00027a040

[5] Wu TF, Orgel LE (1992) Nonenzymatic Template-Directed Synthesis on Hairpin. Oligonucleotides.2. Templates Containing Cytidine and Guanosine Residues. Journal of The American Chemical Society 114: 5496-5501. 11538402 10.1021/ja00040a002

[6] Wu T, Orgel LE (1992) Nonenzymatic Template-Directed Synthesis on Hairpin. Oligonucleotides.3. Incorporation of Adenosine and Uridine Residues. Journal of The American Chemical Society 114: 7963-7969. 11538876 10.1021/ja00047a001

[7] Kurz M, Göbel K, Hartel C, Göbel MW (1998) Acridine-Labeled Primers as Tools for the Study of Nonenzymatic RNA Oligomerization. Helv Chim Acta 81: 1156-1180. doi:10.1002/hlca.19980810528.

[8] Joyce GF (2002) The antiquity of RNA-based evolution. Nature 418: 214-221. doi:10.1038/418214a. PubMed: 12110897. 12110897 10.1038/418214a

[9] Orgel LE (2004) Prebiotic chemistry and the origin of the RNA world. Crit Rev Biochem Mol Biol 39: 99-123. doi:10.1080/10409230490460765. PubMed: 15217990. 15217990 10.1080/10409230490460765

[10] Dörr M, Löffler PMG, Monnard P-A (2012) Non-enzymatic Polymerization of Nucleic Acids from Monomers: Monomer Self- Condensation and Template-Directed Reactions. Curr Org Synth 9: 735-763. doi:10.2174/157017912803901691.

[11] Hey M, Hartel C, Gobel MW (2003) Nonenzymatic oligomerization of ribonucleotides: Towards in vitro selection experiments. Helv Chim Acta 86: 844-854. doi:10.1002/hlca.200390084.

[12] Stribling R, Miller SL (1991) Attempted Nonenzymatic Template-Directed Oligomerizations on a Polyadenylic-Acid Template - Implications for the Nature of the 1st Genetic Material. J Mol Evol 32: 282-288. doi:10.1007/BF02102185. PubMed: 11538258. 11538258 10.1007/BF02102185

[13] Kanavarioti A, Monnard PA, Deamer DW (2001) Eutectic phases in ice facilitate nonenzymatic nucleic acid synthesis. Astrobiology 1: 271-281. doi:10.1089/15311070152757465. PubMed: 12448990. 12448990 10.1089/15311070152757465

[14] Monnard PA, Kanavarioti A, Deamer DW (2003) Eutectic phase polymerization of activated ribonucleotide mixtures yields quasi-equimolar incorporation of purine and pyrimidine nucleobases. J Am Chem Soc 125: 13734-13740. doi:10.1021/ja036465h. PubMed: 14599212. 14599212 10.1021/ja036465h

[15] Monnard PA, Szostak JW (2008) Metal-ion catalyzed polymerization in the eutectic phase in water-ice: A possible approach to template-directed RNA polymerization. J Inorg Biochem 102: 1104-1111. doi:10.1016/j.jinorgbio.2008.01.026. PubMed: 18329104. 18329104 10.1016/j.jinorgbio.2008.01.026

[16] Vogel SR, Richert C (2007) Adenosine residues in the template do not block spontaneous replication steps of RNA. Chem Commun: 1896-1898. 10.1039/b702768k 17695221

[17] Deck C, Jauker M, Richert C (2011) Efficient enzyme-free copying of all four nucleobases templated by immobilized RNA. Nat Chem 3: 603-608. doi:10.1038/nchem.1086. PubMed: 21778979. 21778979 10.1038/nchem.1086

[18] Hud NV, Jain SS, Li X, Lynn DG (2007) Addressing the Problems of Base Pairing and Strand Cyclization in Template-Directed Synthesis. Chem Biodivers 4: 768-783. doi:10.1002/cbdv.200790063. PubMed: 17443888. 17443888 10.1002/cbdv.200790063

[19] Kanavarioti A, Lee LF, Gangopadhyay S (1999) Relative reactivity of ribosyl 2'-OH vs. 3'-OH in concentrated aqueous solutions of phosphoimidazolide activated nucleotides. Orig Life Evol Biosph 29: 473-487. doi:10.1023/A:1006540607594. PubMed: 10573689. 10573689 10.1023/a:1006540607594

[20] Kervio E, Hochgesand A, Steiner UE, Richert C (2010) Templating efficiency of naked DNA. Proc Natl Acad Sci U S A 107: 12074-12079. doi:10.1073/pnas.0914872107. PubMed: 20554916. 20554916 10.1073/pnas.0914872107 PMC2901432

[21] Sawai H (1976) Catalysis of internucleotide bond formation by divalent metal ions. J Am Chem Soc 98: 7037-7039. doi:10.1021/ja00438a050. PubMed: 965662. 965662 10.1021/ja00438a050

[22] Lohrmann R, Bridson PK, Orgel LE (1980) Efficient metal-ion catalyzed template-directed oligonucleotide synthesis. Science 208: 1464-1465. doi:10.1126/science.6247762. PubMed: 6247762. 6247762 10.1126/science.6247762

[23] Trinks H, Schröder W, Biebricher CK (2005) Ice and the origin of life. Orig Life Evolution Biospheres 35: 429-445. doi:10.1007/s11084-005-5009-1. PubMed: 16231207. 10.1007/s11084-005-5009-1 16231207

[24] Inoue T, Orgel LE (1983) A nonenzymatic RNA polymerase model. Science 219: 859-862. doi:10.1126/science.6186026. PubMed: 6186026. 6186026 10.1126/science.6186026

[25] Rajamani S, Ichida JK, Antal T, Treco DA, Leu K et al. (2010) Effect of Stalling after Mismatches on the Error Catastrophe in Nonenzymatic Nucleic Acid Replication. J Am Chem Soc 132: 5880-5885. doi:10.1021/ja100780p. PubMed: 20359213. 20359213 10.1021/ja100780p PMC2857888

[26] Hartel C, Göbel MW (2000) Substitution of adenine by purine-2,6-diamine improves the nonenzymatic oligomerization of ribonucleotides on templates containing thymidine. Helv Chim Acta 83: 2541-2549. doi:10.1002/1522-2675(20000906)83:9.

[27] Thayer JR, Barreto V, Rao S, Pohl C (2005) Control of oligonucleotide retention on a pH-stabilized strong anion exchange column. Anal Biochem 338: 39-47. doi:10.1016/j.ab.2004.11.013. PubMed: 15707934. 15707934 10.1016/j.ab.2004.11.013

[28] Thayer JR, Rao S, Puri N, Burnett CA, Young M (2007) Identification of aberrant 2'-5' RNA linkage isomers by pellicular anion exchange chromatography. Anal Biochem 361: 132-139. doi:10.1016/j.ab.2006.10.032. PubMed: 17161825. 17161825 10.1016/j.ab.2006.10.032

[29] Oligo Analyzer 3.1, Integrated DNA Technologies website. Available: http://eu.idtdna.com/analyzer/applications/oligoanalyzer/default.aspx. Accessed 2013 January 2.

[30] Schrum JP, Ricardo A, Krishnamurthy M, Blain JC, Szostak JW (2009) Efficient and Rapid Template-Directed Nucleic Acid Copying Using 2'-Amino-2',3'-dideoxyribonucleoside-5'-Phosphorimidazolide Monomers. J Am Chem Soc 131: 14560-14570. doi:10.1021/ja906557v. PubMed: 19757789. 19757789 10.1021/ja906557v PMC2759813

[31] Joyce GF, Inoue T, Orgel LE (1984) Non-enzymatic template-directed synthesis on RNA random copolymers. Poly(C, U) templates. J Mol Biol 176: 279-306. doi:10.1016/0022-2836(84)90425-X. PubMed: 6205154. 6205154 10.1016/0022-2836(84)90425-x

[32] Joyce GF, Orgel LE (1986) Non-enzymic template-directed synthesis on RNA random copolymers. Poly(C, G) templates. J Mol Biol 188: 433-441. doi:10.1016/0022-2836(86)90166-X. PubMed: 2426455. 2426455 10.1016/0022-2836(86)90166-x

[33] Joyce GF, Orgel LE (1988) Non-enzymatic template-directed synthesis on RNA random copolymers. Poly(C,A) templates. J Mol Biol 202: 677-681. doi:10.1016/0022-2836(88)90297-5. PubMed: 2459395. 2459395 10.1016/0022-2836(88)90297-5

[34] Kanavarioti A, Bernasconi CF, Doodokyan DL, Alberas DJ (1989) Magnesium ion catalyzed P-N bond hydrolysis in imidazolide-activated nucleotides. Relevance to template-directed synthesis of polynucleotides. J Am Chem Soc 111: 7247-7257. doi:10.1021/ja00200a053. PubMed: 11542186. 11542186 10.1021/ja00200a053

[35] Leu K, Kervio E, Obermayer B, Turk-MacLeod RM, Yuan C et al. (2013) Cascade of Reduced Speed and Accuracy after Errors in Enzyme-Free Copying of Nucleic Acid Sequences. J Am Chem Soc 135: 354-366. doi:10.1021/ja3095558. PubMed: 23259600. 23259600 10.1021/ja3095558 PMC3557965

[36] Attwater J, Wochner A, Pinheiro VB, Coulson A, Holliger P (2010) Ice as a protocellular medium for RNA replication. Nat Communications 1: 76-. PubMed: 20865803. 10.1038/ncomms1076 20865803

[37] Zhao C, Yin RC, Yin JF, Zhang DP, Wang HL (2012) Capillary Monolithic Bioreactor of Immobilized Snake Venom Phosphodiesterase for Mass Spectrometry Based Oligodeoxynucleotide Sequencing. Anal Chem 84: 1157-1164. doi:10.1021/ac2029387. PubMed: 22208283. 22208283 10.1021/ac2029387

[38] Vaish NK, Fraley AW, Szostak JW, McLaughlin LW (2000) Expanding the structural and functional diversity of RNA: analog uridine triphosphates as candidates for in vitro selection of nucleic acids. Nucleic Acids Res 28: 3316-3322. doi:10.1093/nar/28.17.3316. PubMed: 10954600. 10954600 10.1093/nar/28.17.3316 PMC110695

[39] Huang W, Ferris JP (2006) One-step, regioselective synthesis of up to 50-mers of RNA oligomers by montmorillonite catalysis. J Am Chem Soc 128: 8914-8919. doi:10.1021/ja061782k. PubMed: 16819887. 16819887 10.1021/ja061782k

[40] Prabahar KJ, Ferris JP (1997) Adenine derivatives as phosphate-activating groups for the regioselective formation of 3',5'-linked oligoadenylates on montmorillonite: possible phosphate-activating groups for the prebiotic synthesis of RNA. J Am Chem Soc 119: 4330-4337. doi:10.1021/ja9700764. PubMed: 11543599. 11543599 10.1021/ja9700764

[41] Sagan C, Mullen G (1972) Earth and Mars - Evolution of Atmospheres and Surface Temperatures. Science 177: 52–56. doi:10.1126/science.177.4043.52. PubMed: 17756316. 17756316 10.1126/science.177.4043.52

[42] Zahnle K, Arndt N, Cockell CS, Halliday A, Nisbet E et al. (2007) Emergence of a habitable planet. Space Sci Rev 129: 35-78. doi:10.1007/s11214-007-9225-z.

[43] Armstrong RL (1981) Radiogenic Isotopes - the Case for Crustal Recycling on a near-Steady-State No-Continental-Growth Earth. Philos Trans R Soc Lond A Math Phys Eng Sci 301: 443-472. doi:10.1098/rsta.1981.0122.

[44] Iizuka T, Komiya T, Ueno Y, Katayama I, Uehara Y et al. (2007) Geology and zircon geochronology of the Acasta Gneiss Complex, northwestern Canada: New constraints on its tectonothermal history. Precambrian Res 153: 179-208. doi:10.1016/j.precamres.2006.11.017.

[45] Arndt NT, Nisbet EG (2012) Processes on the Young Earth and the Habitats of Early Life. Annu Rev Earth Planet Sci, Vol 40 40: 521-549.

[46] Bada JL, Bigham C, Miller SL (1994) Impact Melting of Frozen Oceans on the Early Earth - Implications for the Origin of Life. Proc Natl Acad Sci U S A 91: 1248-1250. doi:10.1073/pnas.91.4.1248. PubMed: 11539550. 11539550 10.1073/pnas.91.4.1248 PMC43134

[47] Wilde SA, Valley JW, Peck WH, Graham CM (2001) Evidence from detrital zircons for the existence of continental crust and oceans on the Earth 4.4 Gyr ago. Nature 409: 175-178. doi:10.1038/35051550. PubMed: 11196637. 11196637 10.1038/35051550

