# Supplementary material for: Sliding over the Blocks in Enzyme-Free RNA Copying – One-Pot Primer Extension in Ice
Source: PLoS One. 2013 Sep 18;8(9):e75617. doi: 10.1371/journal.pone.0075617 (PMC3776762; doi:10.1371/journal.pone.0075617)

Supporting Information

Sliding over the blocks in enzyme-free RNA copying – one-pot primer extension in ice

Philipp M. G. Löffler[1], Joost Groen[1,2], Mark Dörr[1,3], and Pierre-Alain Monnard[1,]*

[1]Department of Physics, Chemistry and Pharmaceutical Sciences, University of Southern Denmark, Odense, Denmark, [2]Joost Groen, Institute for Molecules and Materials, Radboud University Nijmegen, Nijmegen, Netherlands, [3]Mark Dörr, Department of Biotechnology & Enzyme Catalysis, Ernst-Moritz-Arndt University of Greifswald, Greifswald, Germany

* Email: monnard@sdu.dk

Index

1. Supporting Materials and Methods ... 2
2. Dependence on the presence of metal ions ... 2
3. Monomer compositions for competitive elongation in setup 1 ... 3
4. Mass Spectrometry of selected elongation products ... 5
   4.1 Setup 2 mass spectrometry data ... 5
   4.2 Setup 3 mass spectrometry data ... 7
   4.3 Setup 4 mass spectrometry data ... 10
5. Control reactions for setup 3 and 4 ... 12
6. Enzymatic digestions ... 15
   6.1 Enzymatic digestions after competitive elongation. ... 17
   6.2 Digestions of triply elongated primers ... 18

1. Supporting Materials and Methods

Activation of RNA trimer UUG

Chemicals: 1-[Bis(dimethylamino)methylene]-1H-1,2,3-triazolo[4,5-b]pyridinium-3-oxid hexafluoro-phosphate (HATU), O-(Benzotriazol-1-yl)-N,N,N′,N′-tetramethyluronium hexafluorophosphate (HBTU), Imidazole (Im), N,N-Diisopropylethylamine (DIEA) and dimethylformamide (DMF) purchased from Sigma (all ACS reagent grade). The trimer 5′-phosphate-rUrUrG (pUUG) was purchased from W.M. Keck Biotechnology Resource Laboratory (Yale University, New Haven, USA)

Procedure: pUUG purified by RP-HPLC shortly prior to use (Dionex Acclaim 120 C18; Eluents, **A**: 0.1 M Triethylamine acetate in Millipore H_2O, pH 7; **B**: 50% Acetonitrile in A, flow: 0.75 ml/min; gradient 0%-48% **B** over 20 min.). For the synthesis of the 5′-phosphoramidate each 1.1 mg of HATU and HBTU were dissolved in 10 µl DMF each and both aliquots added to neat 1.2 mg Imidazole. The resulting solution was combined with 1 nmol trimer 5′-pUUG in 10 µl DMF. 10µl DIEA was added to initiate the reaction (final volume 40 µl, 25 µM oligomer). After 30 min reaction time the product, ImpUUG, was isolated from the reaction mixture by RP-HPLC (same gradient as above, later retention time). The collected fraction was lyophilized and stored at -21 °C. Dissolved immediately before use in the trimer ligation control reaction (7.5 µM ImpUUG, 2.5 µM **FP**, 5 µM $\mathbf{t_2}$, 5.2 mM $Mg(NO_3)_2$, 1.2 mM $Pb(NO_3)_2$, 5 mM MES, pH 6.5 in 100 µl Millipore H_2O. Analyzed by anion-exchange HPLC using the same conditions as for the primer extension assays (see main text).

2. Dependence on the presence of metal ions

The non-competetive primer extensions for setup **1** (ImpG/$\mathbf{t_1}$ and ImpU/$\mathbf{t_2}$, see main text Table 2) were tested in the absence of Mg^{2+} and Pb^{2+} ions, where about 1% of primer was elongated with G across the CC-motif and no elongation by U was detected at all across the AA-motif template. Figure S1 below shows the primer conversions after incubation at -18.4 °C with or without metal ions after 2 and 5 days (all other parameters were kept constant)

Figure S1: Control reactions without metal ions. FP elongated with: red – ImpG in presence of $\mathbf{t_1}$, blue – ImpU in presence of $\mathbf{t_2}$. Control reactions performed in absence of $Mg(NO_3)_2$ and $Pb(NO_3)_2$

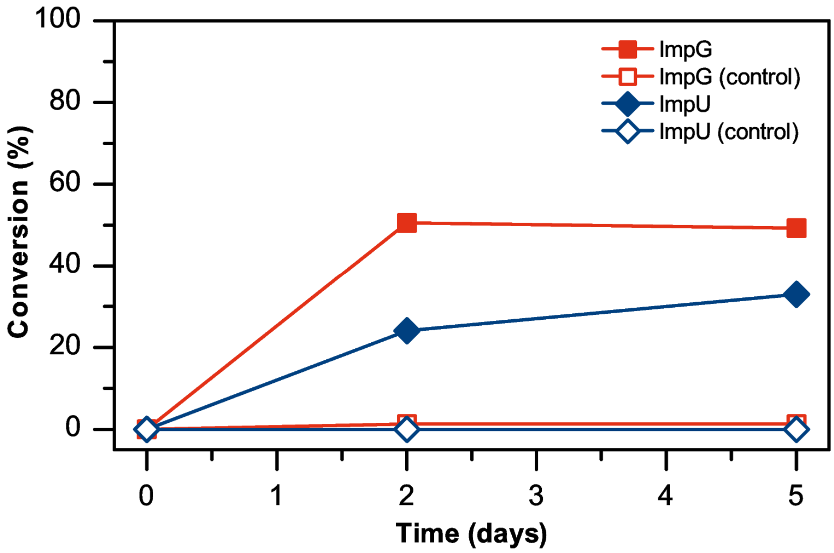

3. Monomer compositions for competitive elongation in setup 1

From earlier results of elongation reactions in the eutectic phase in ice,[1] it has been established that in case of ImpU in presence of the AA template (**t_2**) the best yield is achieved if the monomer is provided at 1.75 mM in the reaction mixture. In case of ImpG that optimal concentration is 0.6mM in presence of a CC template (**t_1**). In the competitive setup **1** using the AA-template these concentrations could either be interpreted as intrinsic to the particular primer/template system or the monomer provided. The following conditions are reported here: i) the total concentration of monomers is 1.75 mM, the optimal concentration observed using only ImpU on its respective template (**t_2**); ii) monomers are supplied at concentrations correspond to their individual optimal concentration, i.e. ImpU at 1.75 mM and ImpG at 0.6 mM; iii) a control experiment, where both monomers were provided at a significantly higher concentration in a 1:1 ratio (2.5 mM ImpU, 2.5 mM ImpG). The results shown in Figure S2, compare the three cases with the non-competitive reactions. In the first case (Figure S2b) clearly the elution pattern of the U reaction is reproduced and at a lower yield. In the second case (Figure S2c) peaks from both individual cases can be observed, including unspecific elongations in the **FP**+1 and +2 region. In the third case (Figure S2d)), the higher concentration let additions of G completely dominates the reaction products, probably due to the stronger stacking interactions of the purine base. Most notably the total yield of the reaction is highest in case ii. (Total primer conversions: i) 23%, ii) 35%, iii) 17%).

Figure S2: Monomer composition in competitive reactions. All analyses where performed after 5 day incubation in the presence of **FP** and $\mathbf{t_2}$, unless indicated otherwise. a) Overlay of the three conditions tested; b) 1:1 ImpU/ImpG; [ImpN] = 1.75mM, in overlay with non-competetive reactions; c) 1.75mM ImpU / 0.6mM ImpG and controls; d) 1:1 ImpU/ImpG, [ImpN] = 5mM.

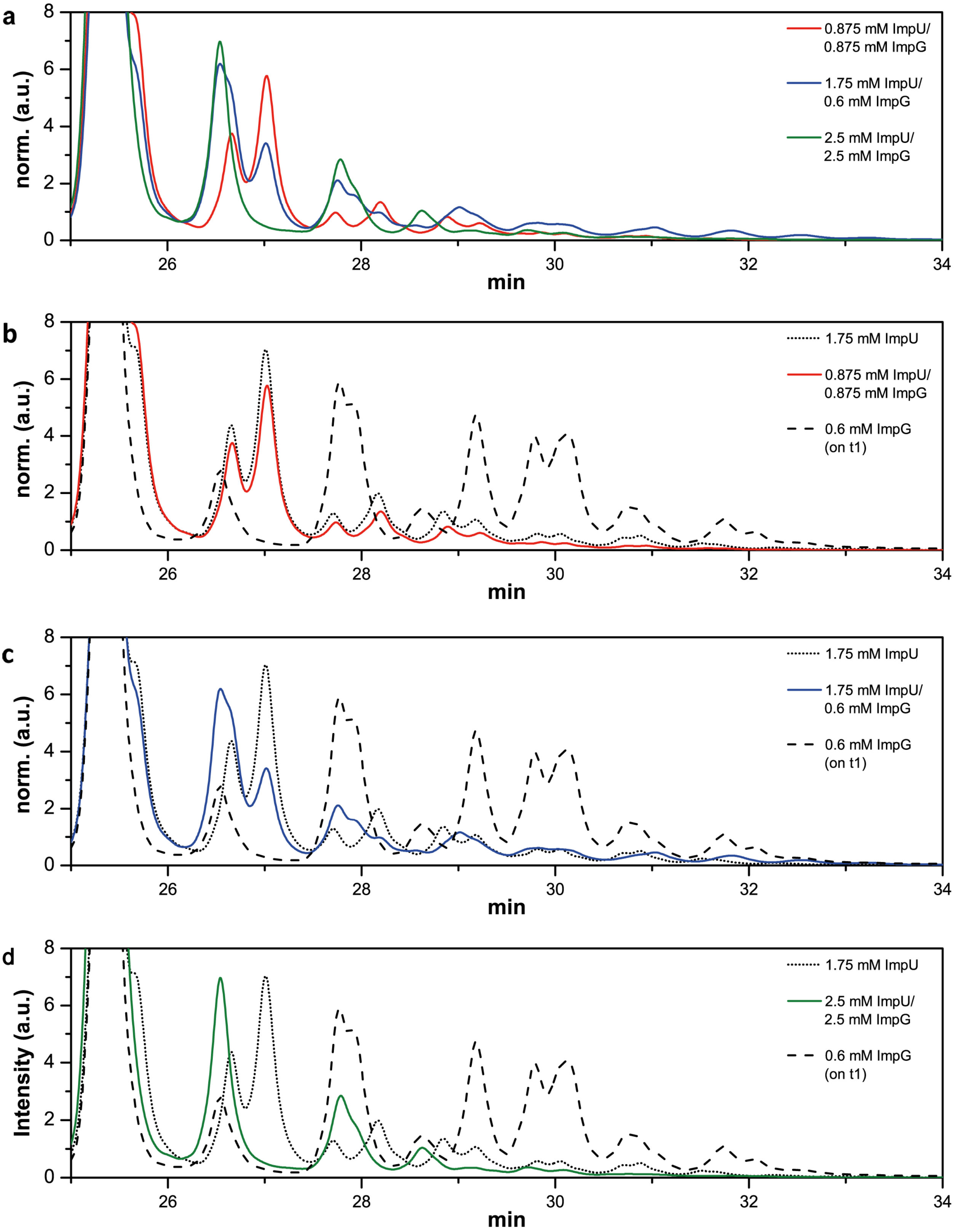

4. Mass Spectrometry of selected elongation products

Products collected from anion-exchange HPLC by fluorescence detection. The chromatograms were obtained using a fresh DNAPac PA 200 column (Method: flow: 1.5 ml/min; solvents: A – 2 mM Trizma, pH 8; B – 250 mM $NaClO_4$, 0-2 min, 0% B; 2-42 min, convex gradient to 65% B, 42-45 min 80% B, λ_{ex} = 494 nm, λ_{ex} = 520 nm). The peaks selected for MS analysis are indicated on the HPLC chromatograms (see below). Each sample was desalted using pipette tips prepared with two layers of 3M Empore C18 material by a standard protocol, followed by evaporation to up-concentrate the samples (> 5 pmol/µl). Aliquots (3µl) of the samples were added to 1.5µl matrix droplets (Matrix: 300 mM trihydroxyacetophenone (THAP) in ethanol, containing 30% v/v 0.1M diammonium citrate in water). Spectra obtained in linear positive mode.

Figures S3, S5 and S7 show the fractions isolated from HPLC, and Figures S4, S6 and S8 the MS spectra matching the indicated fraction counters- Tables S2, S3 and S4 lists the calculated and obtained masses for setups **2**, **3** and **4**, respectively.

4.1 Setup 2 mass spectrometry data

Figure S3: Fractionation of setup 2 products. Analytical HPLC Chromatogram obtained showing the fractions that were later collected for MALDI MS spectrometry (indicated by black bars). The numbers of the fractions correspond to the numbering of MS spectra in Figure S4, where **1** is the primer **FP** and **2-8** are the subsequent elongation products or mixtures of elongation products.

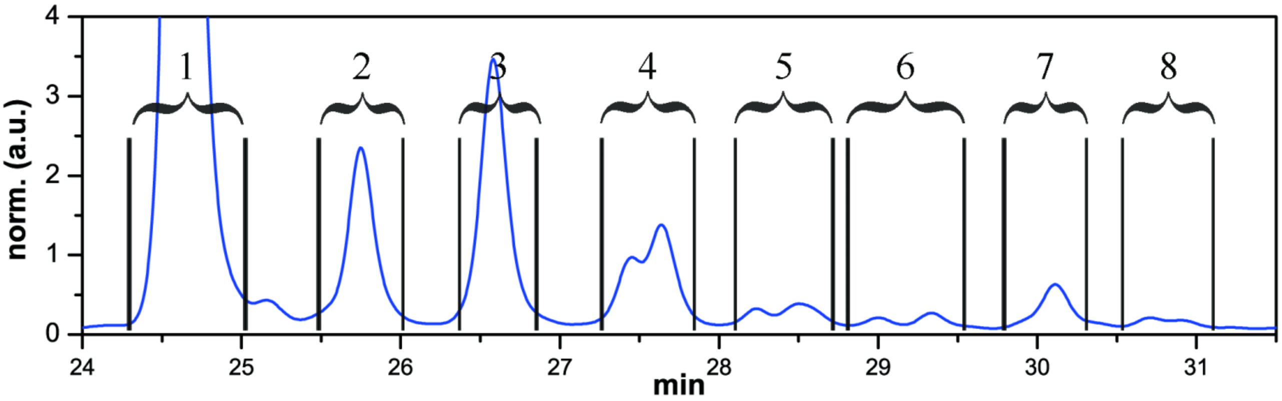

Table S1: Overview of isolated product species for setup 2. For each HPLC fraction of the reaction mixture the observed species are listed. Note that for each molecule at least two regio-isomers exist, that elute differently from HPLC. Regiochemistry indicated if known, otherwise labels (1) and (2) were applied.

Fraction	Species	Molecular mass (g/mol)	Obtained (m/z)
**1**	6-FAM dGdGdA dTdGG (**FP**)	2425.75	2423.45
**2**	**FP**-$^{2'-5'}$U	2731.92	2729.88
**3**	**FP**-$^{3'-5'}$U	2731.92	2731.13
	**FP**-$^{2'-5'}$dG	2754.96	2754.10
**4**	**FP**-$^{3'-5'}$dG	2754.96	2754.7
	**FP**-UU (1)	3038.09	3037.32
**5**	**FP**-UU (2)	3038.09	3036.47
	**FP**-UdG (1)	3061.13	3059.35
**6**	**FP**-UdG (2)	3061.13	3063.37
	**FP**-UUU (1)	3344.26	3346.67
**7**	**FP**-UUU (2, trace)	3344.26	3343.46
	**FP**-UUG (1)	3367.26	3365.86
**8**	**FP**-UUdG (2)	3367.26	3367.63
	**FP**-UUU U (trace)	3650.43	3651.53

Figure S4: MALDI-TOF spectra of the HPLC purified fractions for setup 2. The numbers correspond to the indicated fractions in Figure S5. The detection range of each MS spectrum overlaps with the m/z range of the product ± 1 nt (except for the primer, **FP**). For each peak a concomitant degradation peak at about 151 m/z units below the main peak corresponding to the loss of a guanine base (this peak was annotated in spectrum 2 but omitted in later spectra).

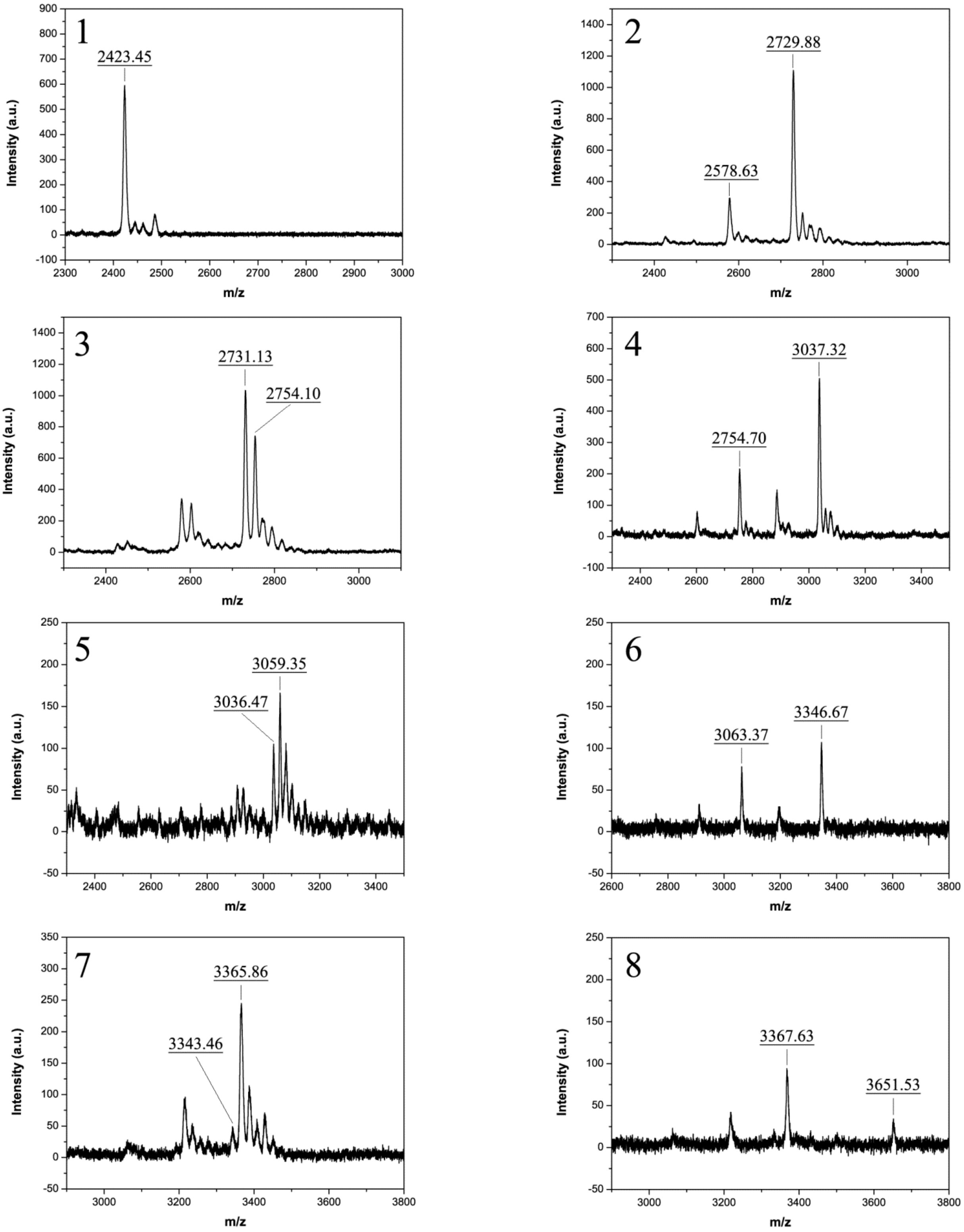

4.2 Setup 3 mass spectrometry data

Figure S5: Fractionation of setup 3 products. Analytical HPLC Chromatogram obtained from setup **3**, showing the fractions that were later collected for MALDI MS spectrometry (indicated by black bars). The numbers of the fractions correspond to the numbering of MS spectra in Figure S6, where **1** is the primer **FP** and **2-11** are the subsequent elongation products or mixtures of elongation products.

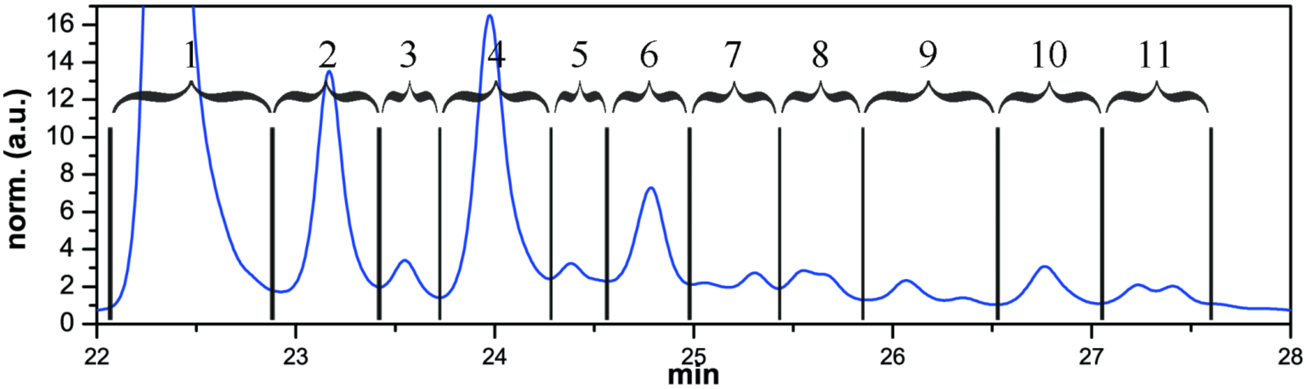

Table S2: Overview of isolated product species of setup 3. For each HPLC fraction of the reaction mixture the observed species are listed. Note that for each molecule at least two regio-isomers exist, that eluted in different HPLC fractions. Major products are labeled (main) while trace products are labeled (trace). Whenever different sequences are possible the elongation is given in brackets with subscripts indicating the abundance of the nucleotide, e.g., **FP**-[U_1A_1] instead of **FP**-UA / FP-AU.

Fraction	Species	Molecular mass (g/mol)	Obtained mass (m/z)
**1**	6-FAM dGdGdA dTdGG (**FP**)	2425.75	2422.54
**2**	**FP-**$^{2'-5'}$U	2731.92	2729.96
**3**	**FP-**$^{2'-5'}$A	2754.99	2753.85
**4**	**FP-**$^{3'-5'}$U (main)	2731.92	2731.97
	**FP-**$^{2'-5'}$dG	2754.96	2754.92
**5**	**FP**-A (main)	2754.99	2753.67
	**FP**-UU	3038.09	3036.92
	**FP**-[U_1A_1] or **FP**-UdG	3061.16	3060.12
**6**	**FP-**$^{3'-5'}$dG (main)	2754.96	2753.70
	**FP**-($^{2'-5'}$U)$_2$	3038.09	3037.14
	**FP**-[U_1A_1] or **FP**-UdG (trace)	3061.16	3060.14
**7**	**FP**-dG (trace)	2754.96	2754.32
	**FP**-UU	3038.09	3036.62
	**FP**-[U1A1] or **FP**-UdG (main)	3061.16	3059.81
**8**	**FP**-[U1A1] or **FP**-UdG (main)	3061.16	3061.23
	**FP**-AA or **FP**-AdG (trace)	3084.23	3083.16
**9**	**FP**-[U_1A_1] or **FP**-UdG (main)	3061.16	3060.02
	**FP**-UUU	3344.26	3043.96
	**FP**-UUdG	3367.26	3366.58
	**FP-[**U_1A_2] (trace)	3390.33	3388.44
**10**	**FP**-UUU (trace)	3344.26	3344.37
	**FP**-UUdG	3367.26	3368.32
	**FP**-UAdG (main)	3390.33	3390.77
**11**	**FP**-UUdG	3367.26	3368.37
	**FP**-UAdG (main)	3390.33	3390.56

Figure S6: MALDI spectrum of the HPLC purified fractions 1-11 of setup 3. The numbers correspond to the indicated fractions in Figure S5. For each peak a concomitant degradation peak at about 151 m/z units below the main peak corresponding to the loss of a guanine base (this peak was annotated in **2** but omitted in later spectra).

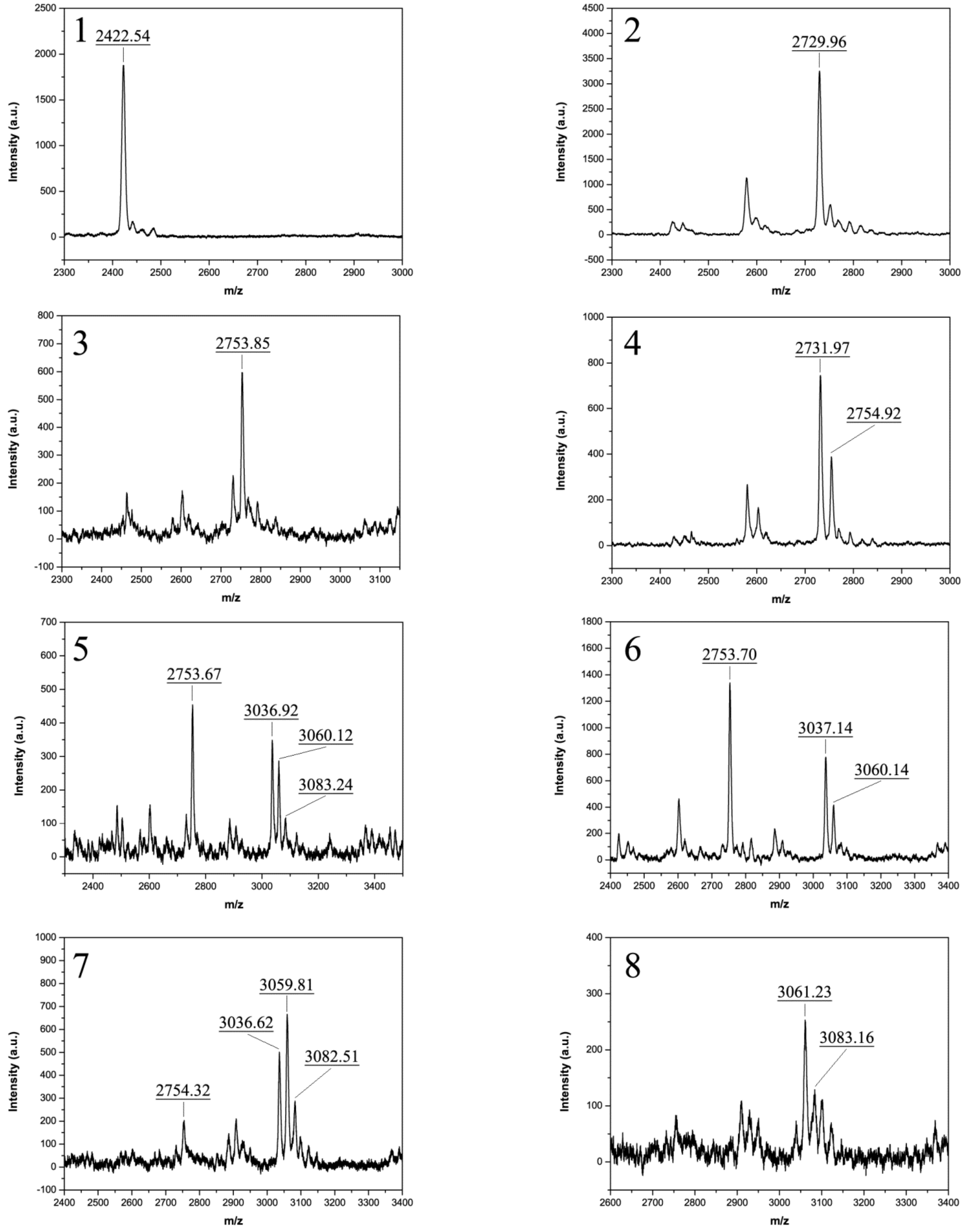

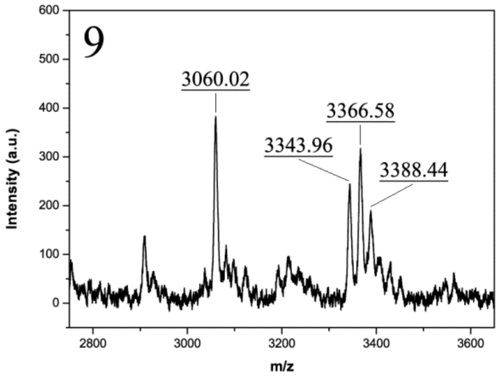
9
3060.02
3366.58
3343.96
3388.44
Intensity (a.u.)
m/z

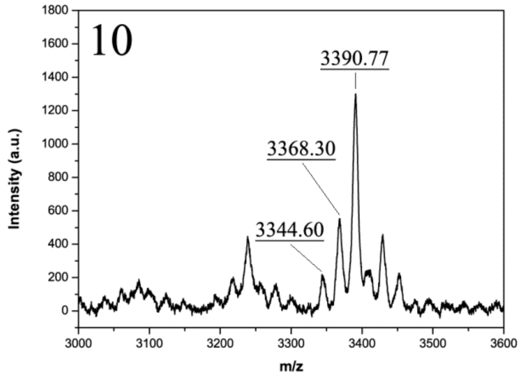
10
3390.77
3368.30
3344.60
Intensity (a.u.)
m/z

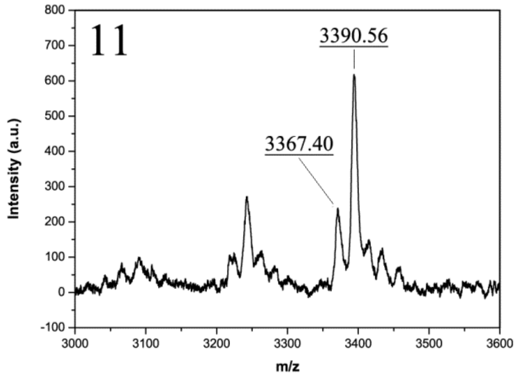
11
3390.56
3367.40
Intensity (a.u.)
m/z

4.3 Setup 4 mass spectrometry data

Figure S7: Fractionation of setup 4 products. Analytical HPLC Chromatogram obtained from setup **4**, showing the fractions that were later collected for MALDI MS spectrometry (indicated by black bars). The numbers of the fractions correspond to the numbering of MS spectra in Figure S8, where **1** is the primer **FP** and **2-8** are the subsequent elongation products or mixtures of elongation reaction mixture the observed species are shown.

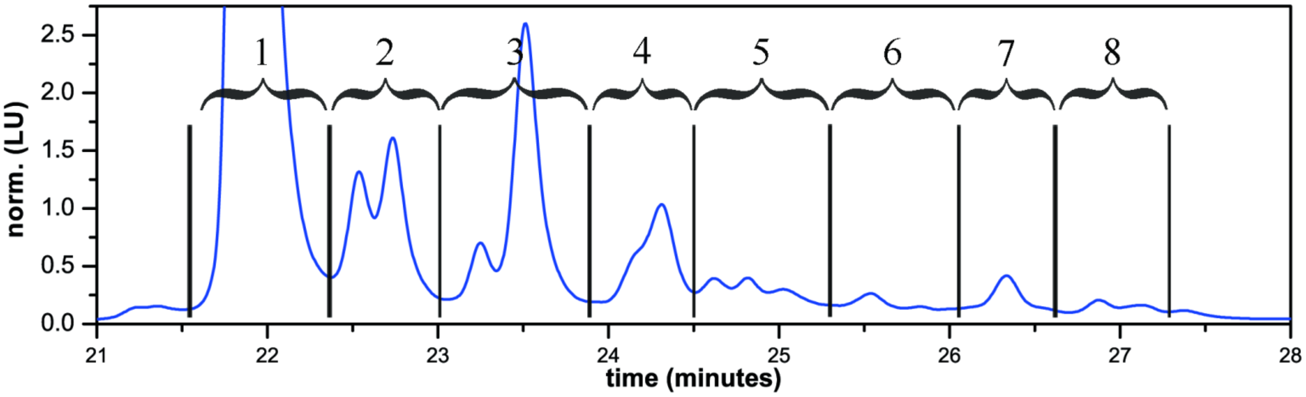

Table S3: Overview of isolated product species of setup 4. For each HPLC fraction of the reaction mixture the observed products and found masses are listed. Note that for each molecule at least two regio-isomers exist, that elute differently from HPLC. Major products are labeled (main) while trace products are labeled (trace). When different sequences are possible, the containing nucleotides are written in square brackets with variables *i* and *j* as counters : **FP**-[X_iY_j], where $i,j = [0..n]$, $i+j = n$, and *n* is the number of inserted monomers. The combinations pertaining to, e.g., **FP**-[U_iC_j], n = 2 are **FP**-UU, **FP**-UC , **FP**-CU or **FP**-CC. In these cases, the molecular masses are given as the average ± $\Delta m/z_{(U\text{-}C)}$ = 0.98.

Fraction	Species	Molecular mass (g/mol)	Obtained mass (m/z)
**1**	6-FAM dGdGdA dTdGG (**FP**)	2425.75	2422.60
**2**	**FP**-$^{2'5'}$C	2730.83	2731.26
	**FP**-$^{2'5'}$U	2731.92	2731.26
**3**	**FP**-$^{3'5'}$C	2730.83	2730.49
	**FP**-$^{3'5'}$U	2731.92	2730.49
	**FP**-$^{2'5'}$dG	2754.96	2752.67
**4**	**FP**-$^{3'5'}$U or **FP**-$^{3'5'}$C (trace)	2731.92 / 2730.83	2730.10
	**FP**-$^{3'5'}$dG	2754.96	2753.43
	**FP**-[U_iC_j], $n = 2$	3037.00 ± 0.98	3036.30
**5**	**FP**-dG (trace)	2754.96	2751.96
	**FP**-[U_iC_j], $n = 2$	3037.00 ± 0.98	3036.66
**6**	Insufficient MALDI sample	N/A	N/A
**7**	**FP**-[U_iC_j]dG, $n = 2$	3366.17 ± 0.98	3365.36
**8**	**FP**-[U_iC_j]dG, $n = 2$	3366.17 ± 0.98	3367.43
	**FP**-[U_iC_j], $n = 4$	3648.25 ± 2 × 0.98	3650.51

Figure S8: MALDI spectrum of the HPLC purified fractions **1**-5, 7 and **8** of setup **4**. The numbers correspond to the indicated fractions in Figure S7. For each peak a concomitant degradation peak at about 151 m/z units below the main peak corresponding to the loss of a guanine base (this peak was annotated in **2** but omitted in later spectra).

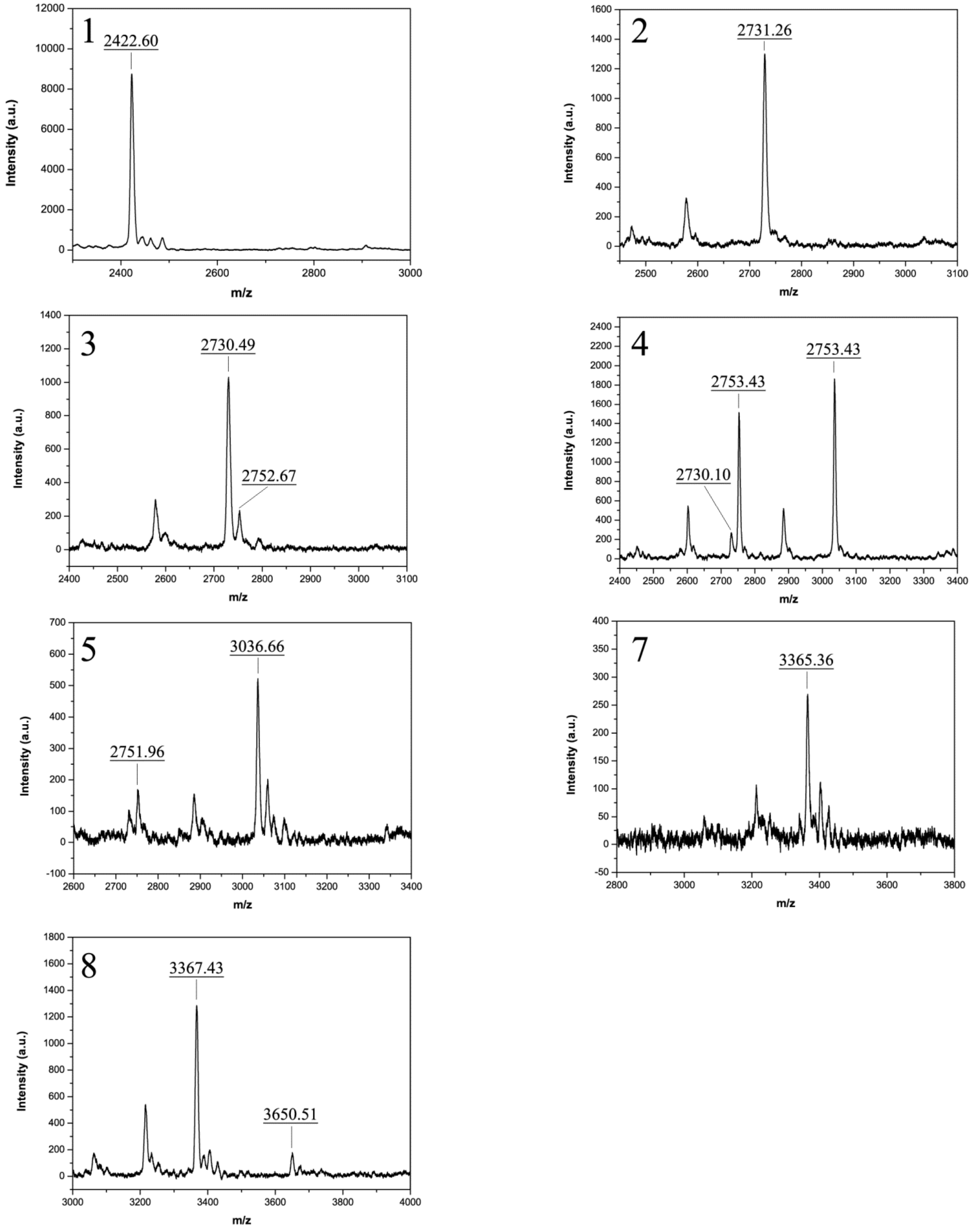

5. Control reactions for setup 3 and 4

As described in the main manuscript, various control reactions (Table S4) were needed to help identifying the **FP**+3 products fromed during primer extensions in setups **3** (Figure S9) and **4** (Figure S10). For each setup an overlay is shown to illustrate which products can be formed if not all nucleotides are present. This allows the identification of **FP**+1 incorporations of the various nucleotides and to compare the unfaithfully formed **FP**+2 and **FP**+3 products to the ones that are observed when the full set of required monomers is supplied. Thus, by removing *pU from the reaction pool, a template mismatch is inevitable if **FP**+1 is formed , i.e., **FP**-Â or **FP**-Ĉ for setup **3** or **4**, respectively. If *pA (setup **3**) or *pC (setup **4**) is removed from the reaction mixture the second insertion will create a mismatch with the template, i.e., on forming **FP**+UÛ across AU or AG, respectively. In all these controls, the amount of formed, bears information about the fidelity of the reaction in the full system, i.e. with all required monomers present. A further control reaction was carried out using **FP-**$^{3'}$U (commercially synthesized oligomer) as a primer, to gauge the efficiency of the reaction after a first correct elongation step. The template motif for the first two insertion in these controls no longer qualifies as “blocking sequence” and accordingly it could be shown that, if the first insertion (U across A) is efficient and correct, the completion to **FP**+3 is also efficient.

Table S4: List of control reactions. All reacted for 14 days, using 2-MeImpN monomers. n/a = not available

**Setup**	**Template** (residues to copy)	**Activated monomer**	**Primer conversion** (%)	**Figure key**
**3**	**t3** (AUC)	U	35	S9, a
		U, A	31	
		A, dG	n/a	S9, b
		U, dG	n/a	S9, c
**3** (**FP-**$^{3'}$U)	**t3** (UC)	A, dG	n/a	S9, d
**4**	**t4** (AGC)	U	31	S10, a
		U, C	34	
		C, dG	n/a	S10, b
		U, dG	n/a	S10, c
**5** (**FP-**$^{3'}$U)	**t3** (GC)	C, dG	n/a	S10, d

Figure S9: Setup 3 control experiments. a) Overlay of the full setup 3 reaction with controls a) with *pU or *pU/*pA only, b) control without *pU, c) control without *pA. d) overlay with the elongation of **FP**-$^{3'}$U by A and dG.

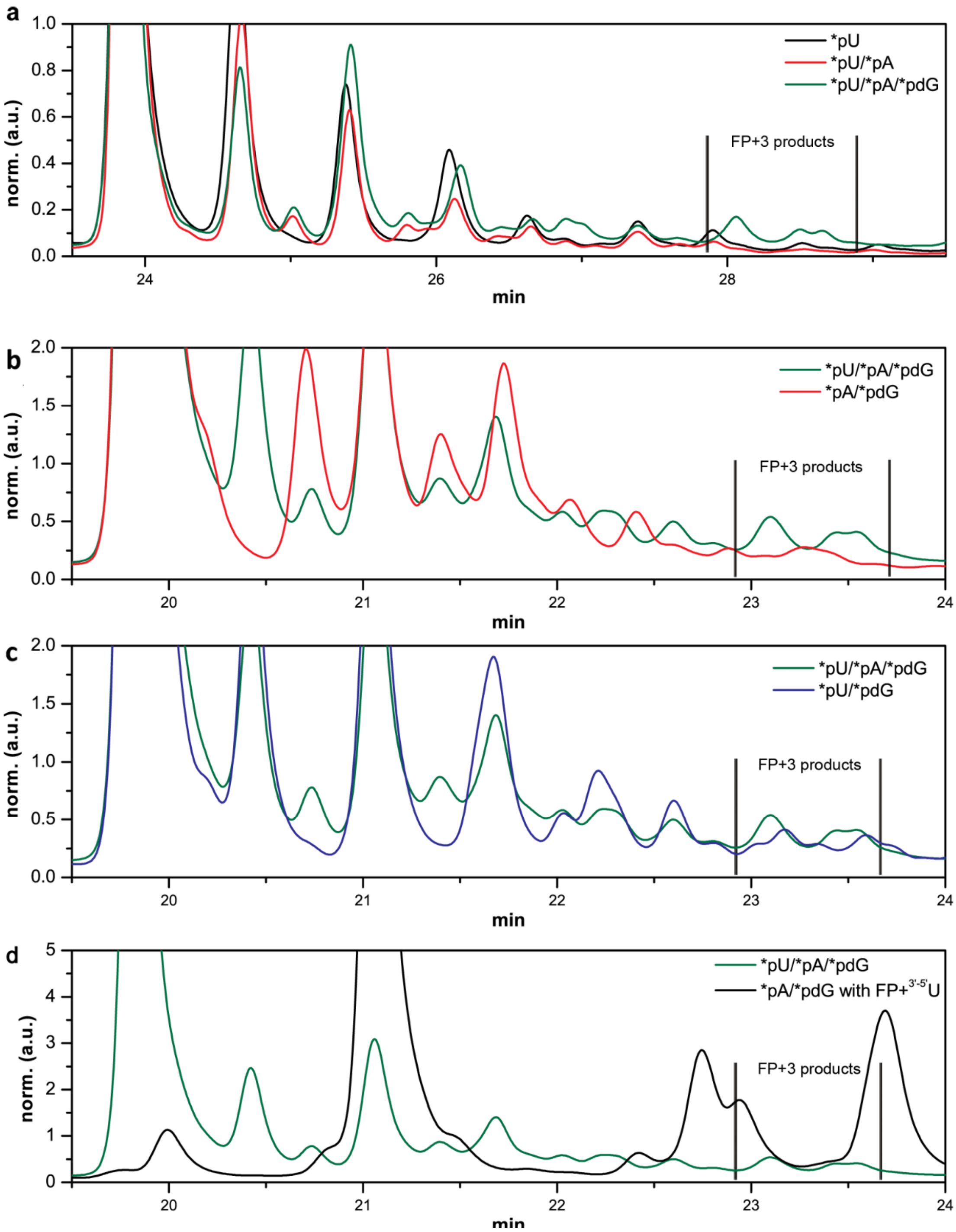

Figure S10: Setup 4 control experiments. Overlay of the full setup 4 reaction with controls a) *pU and *pU/*pC only b) control without *pU. c) control without *pC. d) overlay with the elongation of **FP**-$^{3'}$U by C and dG.

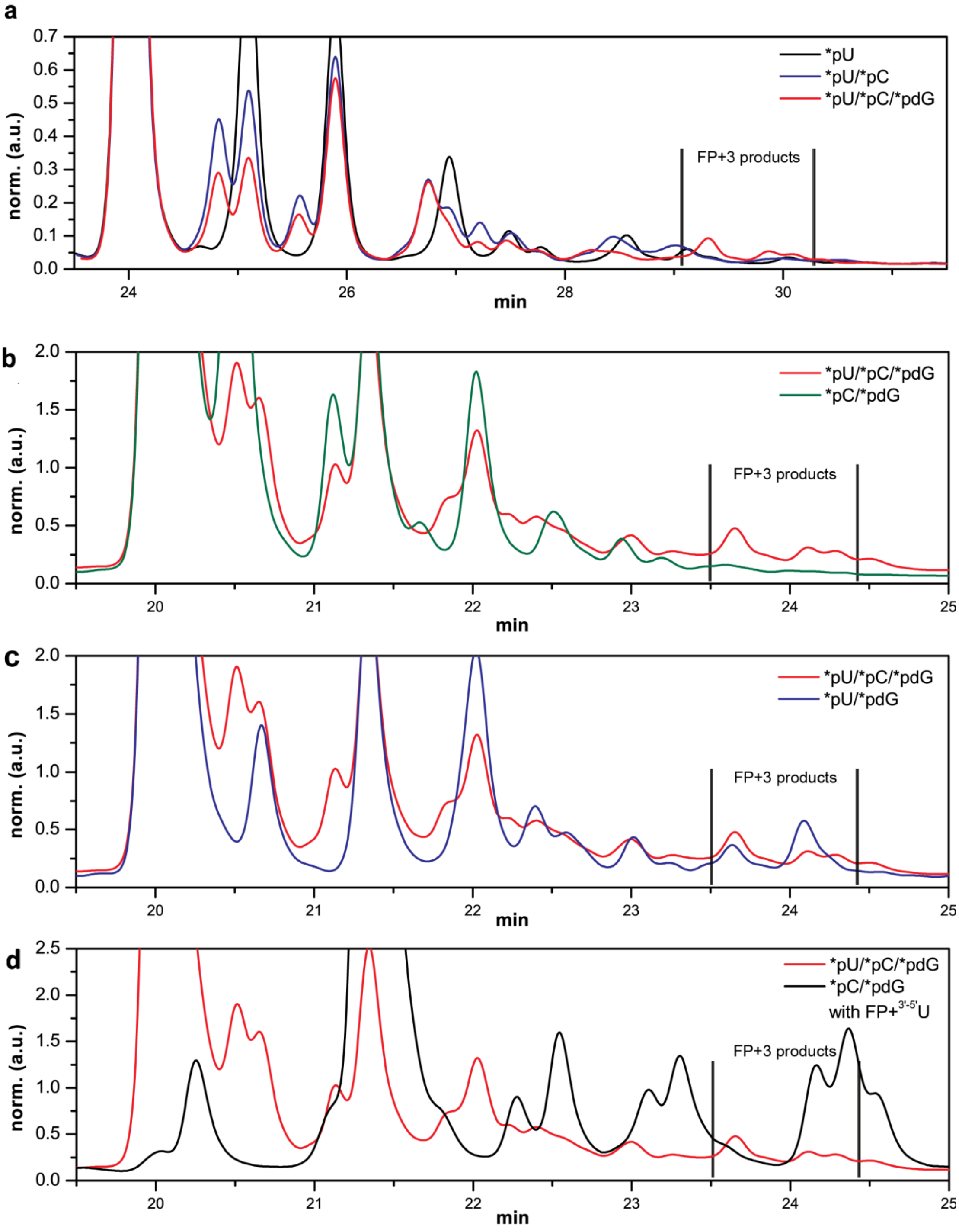

6. Enzymatic digestions

The regioselectivity of the products formed in the non-competetive studies in setups **1** (see Table 1 in the manuscript) was analyzed by enzymatic cleavage selective for 3′-5′-phosphodiester linkages. Figure S11 shows a schematic of this process and illustrates how the cleaved and remaining products were quantified. The amount of 3′-5′ linkages formed in the first elongation was measured by integral of the digestion product (phosphate on the primer, **FP**-p). Aliquots of the reaction mixtures were incubated for 30 min at 37° C with RNAse ONE™ (10u/µl) [1]. Figures S12 and S13 show HPLC chromatograms for ImpG/$\mathbf{t_1}$ and ImpU/$\mathbf{t_2}$, respectively (after incubation with the enzyme (dig.) or without (ref.)). At least three repetitions of each experiment were done. The ratio of the 2′-5′ vs. 3′-5′ regio-isomers for the first nucleotide incorporation was calculated from the difference of the chromatogram integrals after incubation at 37 ˚C of digested vs. control (horizontally shaded area in Figure S11). The incubated control was also compared to the original sample to determine degradation due to temperature.

The digestion allowed assessing the maximal length of products formed (to our limits of sensitivity), as the enzyme does not cleave products with all 2′-5′ connectivity. Consequently, it was observed that the elongation with U (longest observable product, **FP**-(U)$_9$) was less influenced by the presence of a template strand than with G (**FP**-(G)$_6$). Indeed, the yield dropped abruptly after the first non-cognate incorporation at **FP**-(G)$_4$ in case of setup **2**, whereas the yields of **FP**-(U)$_n$ (setup **1**) decayed in an exponential-like fashion from **FP**-(U)$_3$ to **FP**-(U)$_6$.

Figure S11: Schematic illustration of the RNAse digestion. and. The example illustrates the analytical procedure to calculate regioselectivity ratio from the chromatographic traces before (blue, "ref.") and after (red, "dig.") treatment with RNAse. Material consumed is shown by the dark-grey shaded areas. The digestion product (**FP**-p, light-grey shaded area) was quantified to obtain the 3′-5′ to 2′-5′ ratio of the first elongation. If this area corresponds to the total of digested product (dark-grey shaded area), 2′-5′ products exclusively are elongated with the same regiochemistry.

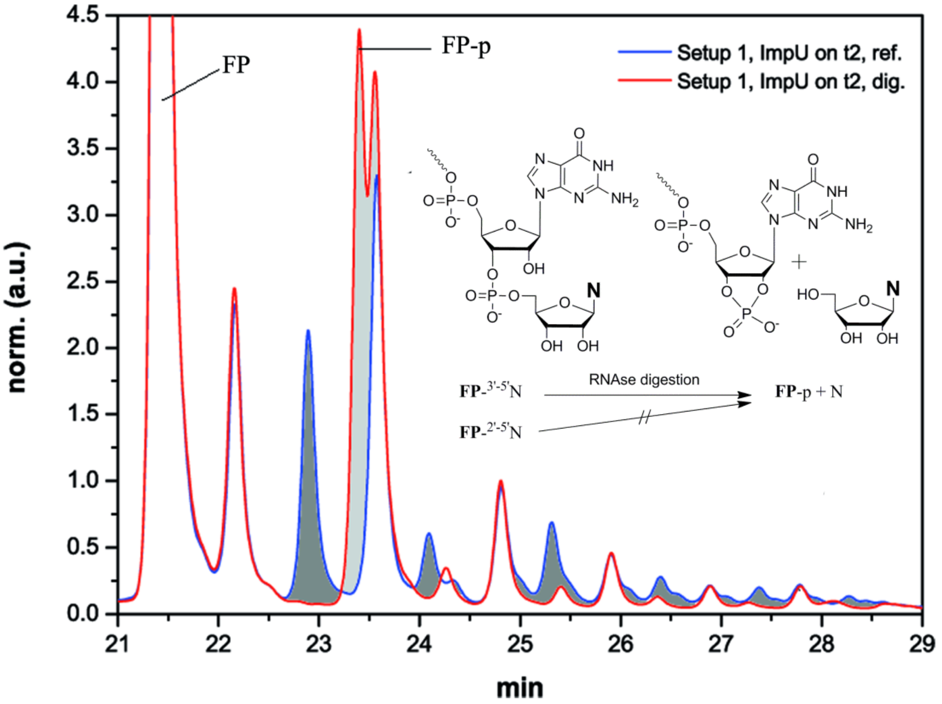

[1] RNAse ONE™ is an endonuclease that cleaves between any two ribonucleotides leading to a 2′-3′-cyclic nucleotide monophosphate These intermediates further hydrolyze to 3′-NMP's.

Figure S12: Enzymatic digestion of elongation products with ImpU in setup 1. FP = fluorescent primer, 6-FAM-dGdGdA dTdGG. **FP-p** denotes the digestion product, see Figure S11. Digestions performed with 20 µl aliquots of 14 day sample

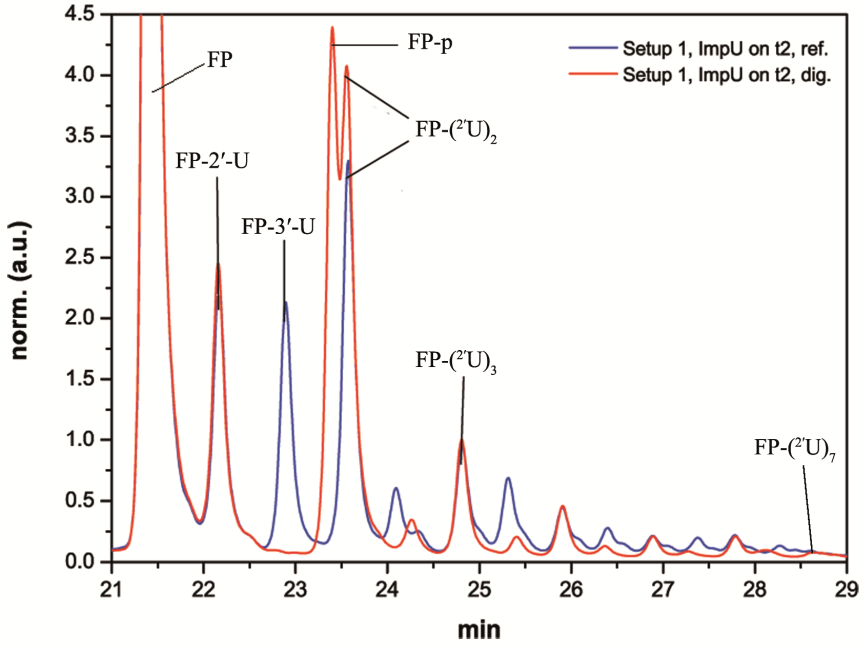

Figure S13: **Enzymatic digestion of elongation products with ImpG in setup 2**. **FP =** fluorescent primer, 6-FAM-dGdGdA dTdGG. **FP-p** denotes the digestion product, see Figure S11. Digestions performed with 20 µl aliquots of 14 day sample

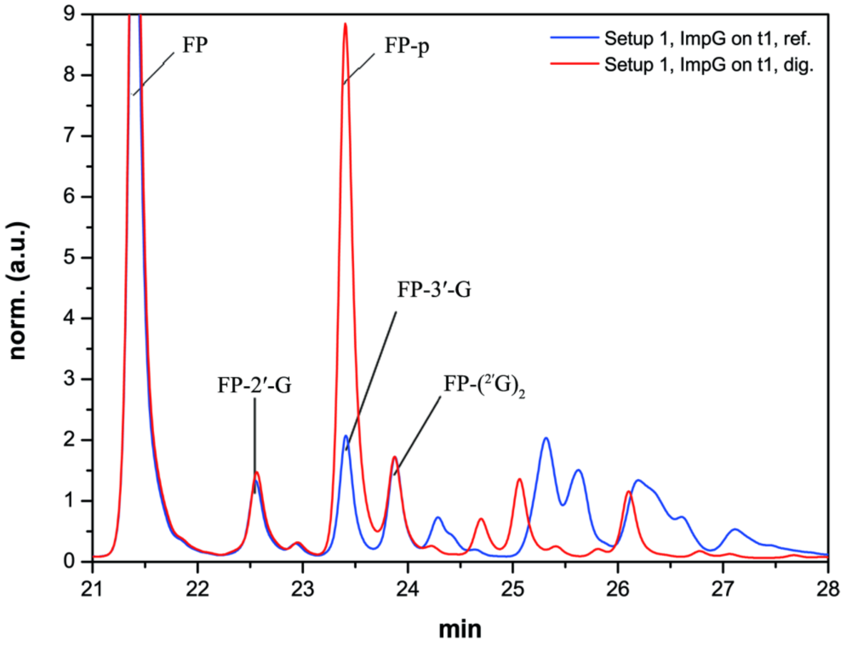

6.1 Enzymatic digestions after competitive elongation.

A typical HPLC analysis for setups **2**, **3** and **4** after 14 days incubation time at -18.4 °C is shown in Figure S14, as the overlay of crude product and degraded with RNAse ONE (as described above).

Figure S14: Enzymatic digestion of crude reaction mixtures of competetive primer extensions. Digestions performed with 20 µl aliquots of 14 day samples from a) setup **2**; b) setup **3**; and c) setup **4**; ref. = untreated, dig. = digested.

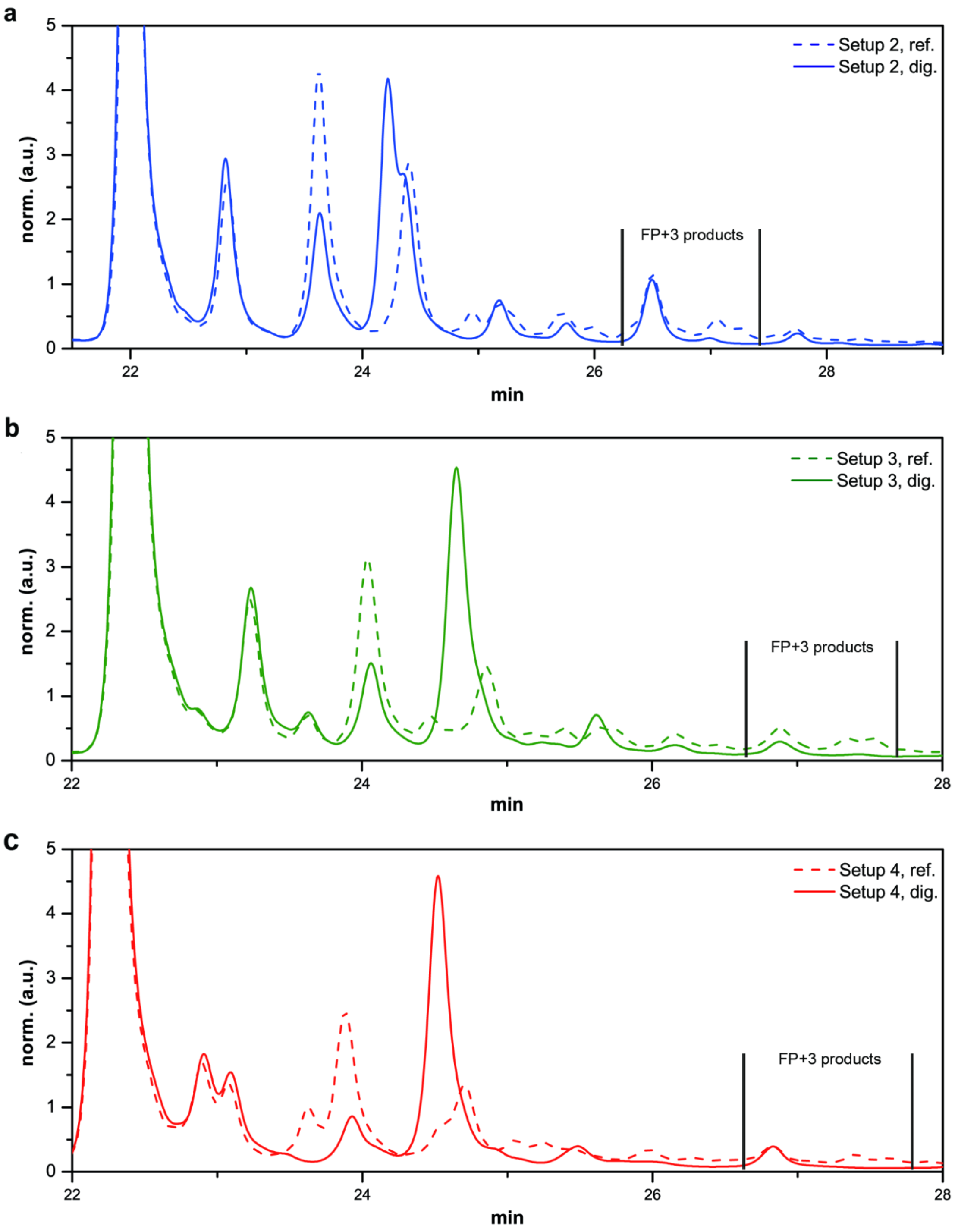

6.2 Digestions of triply elongated primers

By isolating **FP**+3 prodcts and subjecting them to RNAse treatment, it became possible to analyse the digestion products of the triply elongated primers. These products would otherwise co-elute with, e.g. **FP**+1 and **FP**+2 strands. For the reported data, please note the following degradation schemes ($^{2'}$ = 2′-5′-, $^{3'}$ = 3′-5′-linkage, ^{x} = both regioisomers are possible educts):

FP-$^{3'}$N^{x}N^{x}N → **FP**-p

FP-$^{2'}$N$^{3'}$N^{x}N → **FP**-$^{2'}$N-p

FP-$^{2'}$N$^{2'}$N$^{3'}$N→ **FP**-$^{2'}$N$^{2'}$N -p

FP-$^{2'}$N$^{2'}$ N$^{2'}$N remains uncleaved.

Table S5 corresponds to Table 4 in the main manuscript, with addition of breaking down the **FP**-$^{2'}$N^{x}N^{x}N group of regiomers into specific regiomers.

Table S5: Detailed abundances of regioisomers in triply elongated primers.

**Setup**	**Fraction**	**Relative abundances (to total FP+3 product) of**				
		**FP**-$^{3'}$N^{x}N^{x}N (%)	**FP**-$^{2'}$N^{x}N^{x}N (%)	Subdivided in		
(template, monomers)				**FP**-$^{2'}$N$^{3'}$N^{x}N (%)	**FP**-$^{2'}$N$^{2'}$N$^{3'}$N (%)	**FP**-$^{2'}$N$^{2'}$N$^{2'}$N (%)
**2** (t_2, U/**d**G)	I	8	60	4	4	52
	II	23	8	1	1	6
	**total**	**32**	**68**	**5**	**5**	**58**
**3** (t_3, U/A/**d**G)	I	22	28	7	n/a	21
	II	35	15	4	3	8
	**total**	**57**	**43**	**11**	**3**	**29**
**4** (t_4, U/C/**d**G)	I	19	46	6	n/a	40
	II	28	7	1	<0.5	6
	**total**	**47**	**53**	**7**	<0.5	**46**

n/a: not available due to co-elution of the digestion products of **FP**-$^{2'}$N$^{2'}$N$^{3'}$N and **FP**-$^{2'}$N$^{2'}$N$^{2'}$N.

Figure S15 Overlay of digestion products of the indicated fractions (i.e. I and II) with untreated controls; ref. = untreated, dig. = digested.

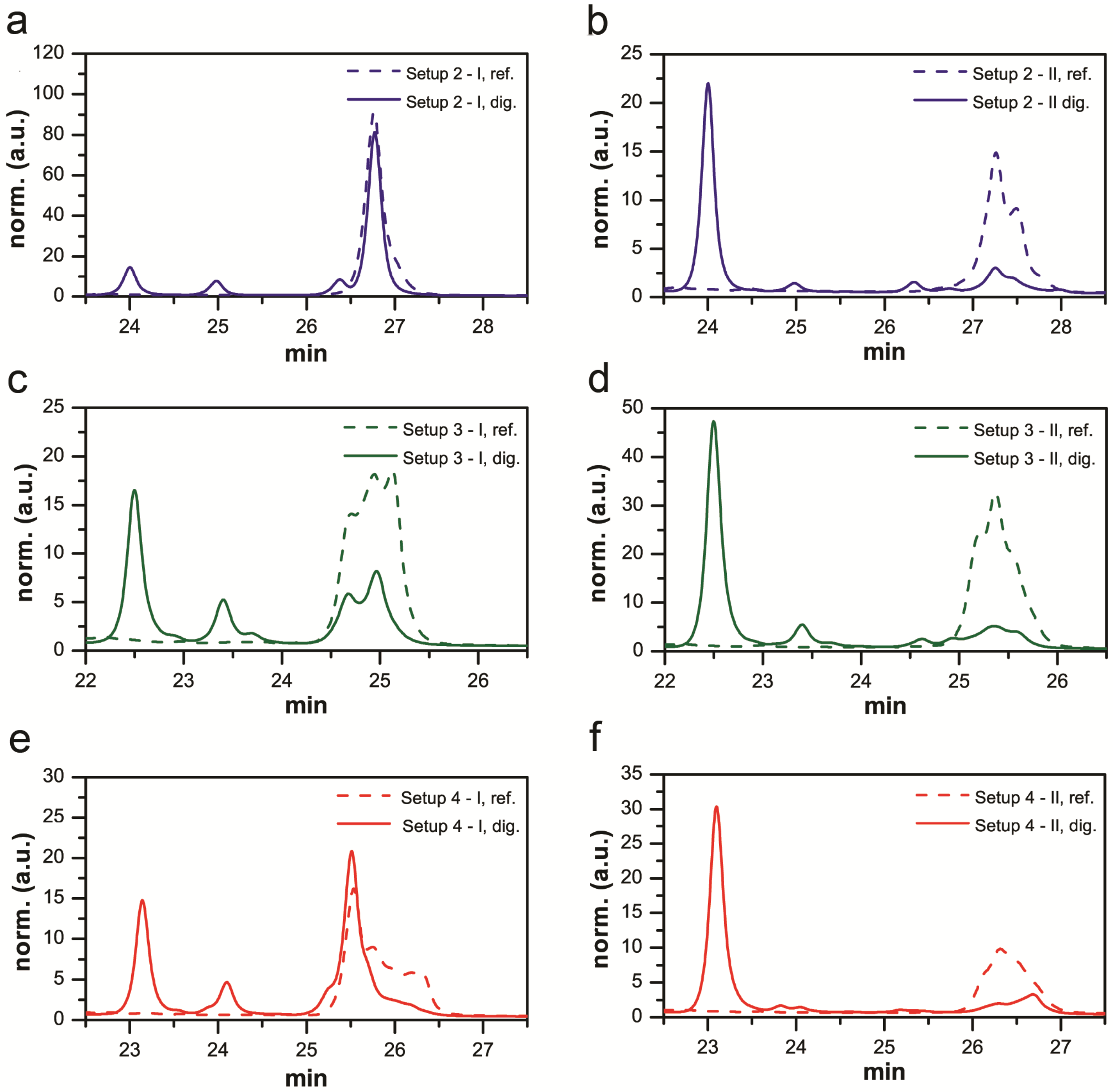

The **FP**-$^{2'}$N-p products were of special interest insofar as it should reveal misincorporations in the first extension step amongst **FP**+3 products. To verify that **FP**-2′-C-p eluted before **FP**-2′-U-p (as does **FP**-C vs. **FP**-U), digestion products from a control experiment (*pC and *pdG across $\mathbf{t_4}$), were used to give a reference for **FP**-2′-C-p (data not shown).

Figure S16: RNAse digestions of isolated FP+3 fractions. Digestions performed with isolated **FP**+3 material setup **3** and **4** reactions after 14 days. (A) Overlay of digestion products the fractions I; (B), analogous overlay for the fractions II. **FP**-$^{2'}$N-p products are annotated; later eluting material consists of small amounts of **FP**-$^{2'}$N$^{2'}$N-p partially superimposing with **FP**-$^{2'}$N$^{2'}$N$^{2'}$N extension products (resistant to digestion).

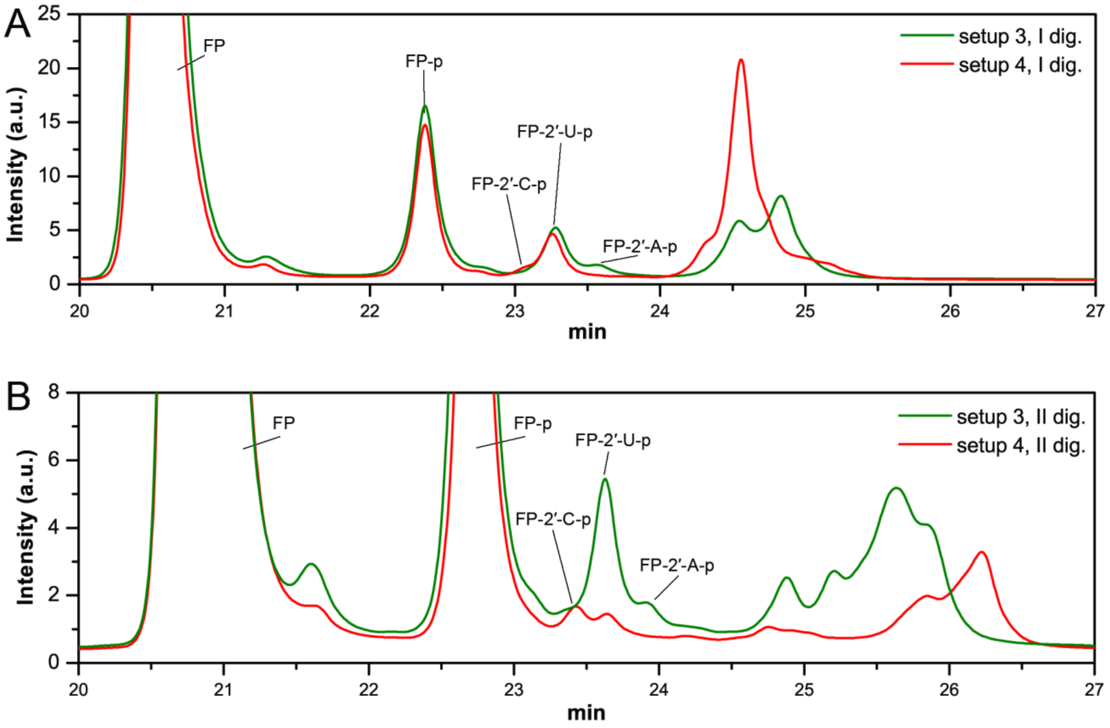

Supplement: File S1 — Combined Supporting Information document containing Supporting Materials and Methods, Tables S1-S5 and Figures S1-S16. (PDF) [file pone.0075617.s001.pdf]
